# A large-scale CRISPR screen reveals context-specific genetic regulation of retinal ganglion cell regeneration

**DOI:** 10.1242/dev.202754

**Published:** 2024-08-12

**Authors:** Kevin Emmerich, John Hageter, Thanh Hoang, Pin Lyu, Abigail V. Sharrock, Anneliese Ceisel, James Thierer, Zeeshaan Chunawala, Saumya Nimmagadda, Isabella Palazzo, Frazer Matthews, Liyun Zhang, David T. White, Catalina Rodriguez, Gianna Graziano, Patrick Marcos, Adam May, Tim Mulligan, Barak Reibman, Meera T. Saxena, David F. Ackerley, Jiang Qian, Seth Blackshaw, Eric Horstick, Jeff S. Mumm

**Affiliations:** ^1^ Wilmer Eye Institute and the Department of Ophthalmology, Johns Hopkins University School of Medicine, Baltimore, MD 21287, USA; ^2^ McKusick-Nathans Institute and the Department of Genetic Medicine, Johns Hopkins University School of Medicine, Baltimore, MD 21287, USA; ^3^ Department of Biology, West Virginia University, Morgantown, WV 26505, USA; ^4^ Department of Ophthalmology and Visual Sciences, University of Michigan School of Medicine, Ann Arbor, MI 48105, USA; ^5^ Department of Cell and Developmental Biology, University of Michigan School of Medicine, Ann Arbor, MI 48105, USA; ^6^ School of Biological Sciences, Victoria University of Wellington, Wellington 6012, New Zealand; ^7^ Institute for Cell Engineering, Johns Hopkins University School of Medicine, Baltimore, MD 21287, USA; ^8^ Department of Neurology, Johns Hopkins University School of Medicine, Baltimore, MD 21287, USA; ^9^ Department of Neuroscience, Johns Hopkins University School of Medicine, Baltimore, MD 21287, USA; ^10^ Department of Neuroscience, West Virginia University, Morgantown, WV 26506, USA; ^11^ Center for Nanomedicine, Johns Hopkins University School of Medicine, Baltimore, MD 21287, USA

**Keywords:** Ascl1, CRISPR screen, Neural stem cell, Regeneration, Retina

## Abstract

Many genes are known to regulate retinal regeneration after widespread tissue damage. Conversely, genes controlling regeneration after limited cell loss, as per degenerative diseases, are undefined. As stem/progenitor cell responses scale to injury levels, understanding how the extent and specificity of cell loss impact regenerative processes is important. Here, transgenic zebrafish enabling selective retinal ganglion cell (RGC) ablation were used to identify genes that regulate RGC regeneration. A single cell multiomics-informed screen of 100 genes identified seven knockouts that inhibited and 11 that promoted RGC regeneration. Surprisingly, 35 out of 36 genes known and/or implicated as being required for regeneration after widespread retinal damage were not required for RGC regeneration. The loss of seven even enhanced regeneration kinetics, including the proneural factors *neurog1*, *olig2* and *ascl1a*. Mechanistic analyses revealed that *ascl1a* disruption increased the propensity of progenitor cells to produce RGCs, i.e. increased ‘fate bias’. These data demonstrate plasticity in the mechanism through which Müller glia convert to a stem-like state and context specificity in how genes function during regeneration. Increased understanding of how the regeneration of disease-relevant cell types is specifically controlled will support the development of disease-tailored regenerative therapeutics.

## Teaser

**Highlighted Article:** The identification of 18 genes that regulate retinal ganglion cell regeneration reveals that, owing to a lack of concordance with widespread retinal injury paradigms, some genes function in a context-specific manner during regeneration.

## INTRODUCTION

Insights into the genes that regulate retinal regeneration in zebrafish have come almost exclusively from acute widespread retinal injury paradigms, such as puncture wounds (Sharma and Ramachandran, 2022), light damage (LD; Lahne et al., 2020; Lenkowski and Raymond, 2014) and chemical toxins (e.g. NMDA; Hoang et al., 2020). A recent report has clarified that both LD and NMDA are not specific to the targeted cell layer but, like puncture wounds, result in substantial cell death throughout all retinal cell layers (Lyu et al., 2023). Thus, current understanding of the factors controlling retinal regeneration must be framed in the context of replacing the retina as a tissue. In contrast, factors controlling regeneration after selective retinal cell loss, which is a hallmark of retinal degenerative disease, are largely unknown. We posited that accounting for how disease-relevant parameters, such as the extent and specificity of retinal cell death, impact the regenerative process will be important for the development of disease-tailored regenerative therapeutics. Accordingly, we designed a study to identify genes that regulate the regeneration of retinal ganglion cells (RGCs), the cells lost in glaucoma, after selective RGC loss.

Upon an ill-defined threshold of acute retinal damage and/or cell loss, zebrafish Müller glia (MG) cells dedifferentiate to a stem-like state (Bernardos et al., 2007; Fausett et al., 2008; Fimbel et al., 2007) and divide asymmetrically to produce a Muller glia-derived progenitor cell (MGPC; Bernardos et al., 2007). MGPCs then proliferate before differentiating to replace lost cells (Nagashima et al., 2013). A recent comparative study of two widespread retinal damage paradigms (LD and NMDA) in zebrafish revealed the regenerative process is exquisitely tuned to the nature of the retinal injury, showing that MGPC proliferation rates and cell fate decisions were directly correlated to the numbers and types of cell lost (Lyu et al., 2023). Similarly, previous studies of selective retinal cell ablation paradigms had shown that MGPCs can exhibit fate bias, i.e. preferentially giving rise to the lost retinal cell type (D'Orazi et al., 2020; Fraser et al., 2013; Lyu et al., 2023; Ng Chi Kei et al., 2017). In a transcriptomic comparison of two selective cell ablation paradigms, we showed that the majority of gene changes were specific to each model rather than shared across paradigms (Emmerich et al., 2023b). These findings raise the possibility that mechanisms governing retinal regenerative processes are context specific; however, this has yet to be rigorously tested.

To explore mechanisms regulating regeneration in the context of selective retinal cell loss, we developed a transgenic line enabling inducible and selective RGC ablation. Single cell transcriptomics and large-scale genetic screening were then used to identify genes whose disruption altered RGC regeneration kinetics. We found the following: (1) largely unique transcriptomic signatures of reactive MG signatures after RGC ablation versus widespread damage paradigms; (2) strong evidence of MGPC bias toward the RGC fate; and (3) 18 genes whose disruption altered RGC regeneration kinetics. In a test of context specificity, nearly all of the 36 genes previously shown and/or implicated as regulators of retinal regeneration after widespread tissue damage were not required for RGC regeneration. Intriguingly, knockout (KO) of several factors thought to be absolutely required for retinal regeneration actually accelerated RGC regeneration kinetics, including loss of *ascl1a*.

We were particularly intrigued by the *ascl1a* result, as forced expression of Ascl1 in mouse MG cells leads to the production of new neurons after retinal damage (Jorstad et al., 2017; Ueki et al., 2015). Unfortunately, Ascl1 expression alone does not promote substantial production of the retinal cell types most relevant to disease, i.e. RGCs and photoreceptors. However, more recently, overexpression of Ascl1 and Atoh1 was shown to be sufficient for mouse MG producing ‘RGC-like’ cells, i.e. exhibiting some features of RGCs, such as production of action potentials, but sharing a transcriptomic signature with retinal progenitors and early amacrine cells, indicating a lack of full RGC maturation (Pavlou et al., 2024; Todd et al., 2021, 2022). These data show that cell fate can be modulated to promote the regeneration of disease-relevant cell types in the mammalian retina, and highlight the need to better understand how proliferation and cell fate are controlled during retinal regeneration. In mechanistic analyses, we determined that *ascl1a* KO had no effect on proliferation, rather it enhanced MGPC bias towards the RGC fate. These data demonstrate: (1) an underappreciated plasticity in the mechanisms controlling MG dedifferentiation to a stem-like state; and (2) that the molecular regulation of retinal regeneration is context specific; i.e. divergent across paradigms due to the regenerative process being informed by, and actively adapting to, the nature of the retinal injury and/or cell loss. By extension, our results suggest cell-specific regeneration paradigms will help to advance strategies for selectively regenerating disease-relevant cell types.

## RESULTS

### A novel zebrafish RGC regeneration paradigm

**Fig. 1.**
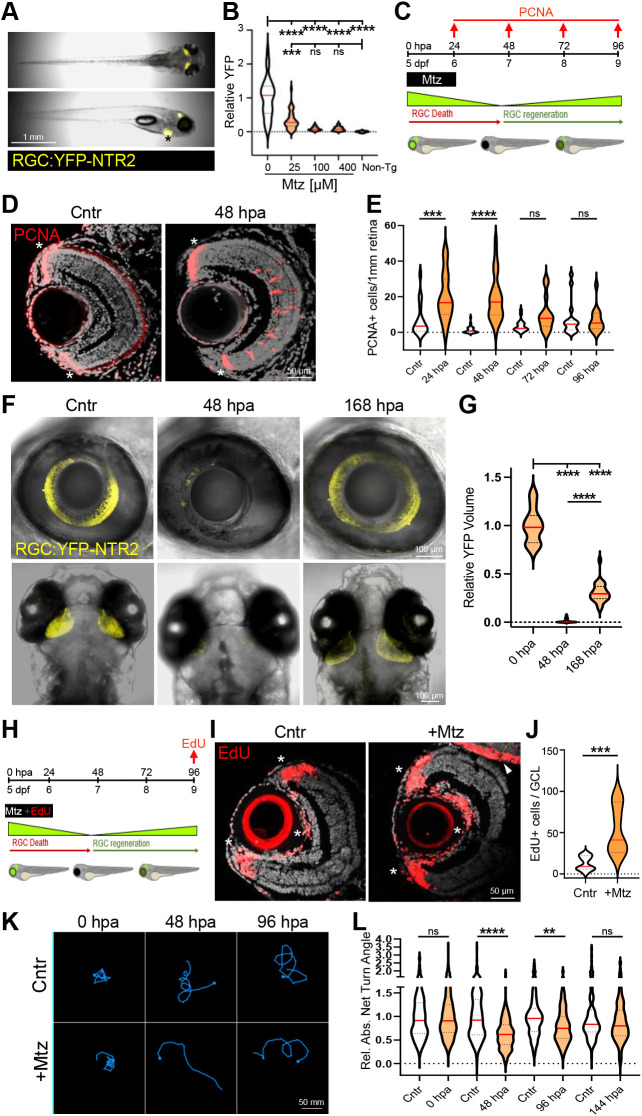
**Characterization of RGC:YFP-NTR2 paradigm.** (A) Whole-fish view of RGC:YFP-NTR2 expression specificity (asterisk indicates zc65-associated myl7:TagRFP ‘tracer’ reporter expression in the heart, not YFP-NTR2 expression). (B) Plate reader-based quantification of YFP loss at 7 dpf (48 hpa) after a 5-6 dpf exposure to Mtz at the indicated concentrations. (C) Experimental setup for analyzing proliferative responses (PCNA immunolabeling) to RGC ablation. (D) Representative PCNA immunostaining (red cells) in the INL and ONL of RGC:YFP-NTR2 retinas treated with or without Mtz (asterisks indicate constitutive proliferation in CMZ). (E) Quantification of PCNA^+^ cells in the INL at 24, 48, 72 and 96 hpa. (F) Representative confocal images of RGC soma and axonal loss (48 hpa) and regeneration (168 hpa) in the retina (top) and tectum (bottom). (G) Imaris-based volumetric quantification of RGC axon loss and regeneration (YFP) from confocal *z*-stacks of the tectum. (H) Experimental setup of EdU-based lineage tracing of early retinal progenitors (24 h pulse, 96 hpa chase). (I) Representative images of EdU immunostaining in unablated (Cntr) and RGC-ablated (+Mtz) RGC:YFP-NTR2 larvae (asterisks indicate CMZ cells in the periphery and endothelial cells near the optic nerve; arrowhead marks EdU^+^ cells in the brain, none of which were included in the quantification). (J) Quantification of EdU^+^ cells in the GCL. (K) Representative swim traces of visual responses to a lights-off stimulus in control and RGC-ablated (+Mtz) larvae at 0, 48 and 96 hpa. (L) Quantification of visual responses in control and RGC-ablated larvae at 0, 48, 96 and 144 hpa. All white violin plots are unablated control larvae (Cntr), all orange violin plots are RGC-ablated larvae (+Mtz). Pair-wise and multiple comparisons are indicated by single lines and extended lines, respectively. **P≤0.01, ***P≤0.001, ****P≤0.0001; ns, not statistically significant.

To investigate factors regulating RGC regeneration, we established transgenic zebrafish enabling prodrug-inducible selective RGC ablation. In this ‘RGC:YFP-NTR2’ line, a yellow fluorescent protein (YFP) reporter and improved bacterial nitroreductase (NTR 2.0) (Sharrock et al., 2022) enzyme are co-expressed exclusively in >95% of RGCs (Fujimoto et al., 2011; Kölsch et al., 2021) (Fig. 1A; Fig. S1A). This allows NTR-expressing RGCs to be ablated upon exposure to NTR prodrug substrates, such as metronidazole (Mtz). To establish an RGC ablation paradigm amenable to large-scale screening, we exposed 5-day post-fertilization (dpf) RGC:YFP-NTR2 larvae to a range of Mtz concentrations and quantified YFP levels at 7 dpf using an established fluorescence plate reader assay system (Walker et al., 2012; White et al., 2017). Mtz treatments of ≥100 μM for 24 h (Fig. 1B) and 48 h (Fig. S1B) were sufficient to reduce YFP expression to non-transgenic (non-Tg) control levels. Accordingly, all subsequent assays used a 24 h 100 μM Mtz treatment to induce maximal RGC loss. To assess whether reductions in YFP correlated to RGC loss, nuclei in the ganglion cell layer (GCL) were quantified in retinal sections of control and Mtz-treated larvae at 7 dpf. Mtz caused an ∼75% reduction in GCL nuclei (Fig. S1C,D), with many remaining GCL nuclei likely being displaced amacrine cells (Zhou et al., 2020).

Extensive acute retinal damage is known to induce Müller glia (MG) to dedifferentiate to a stem-like state (Bernardos et al., 2007; Fausett et al., 2008; Fimbel et al., 2007) and to divide asymmetrically to produce a MG-derived progenitor cell (MGPC; Bernardos et al., 2007). MGPCs are transit amplifying neural progenitor cells that divide further and differentiate into new retinal neurons (Goldman, 2014; Lahne et al., 2020; Nagashima et al., 2013). Accordingly, we asked whether RGC loss induced MG/MGPC proliferation. After Mtz-induced ablation at 5 dpf, RGC:YFP-NTR2 larvae were collected daily until 9 dpf (24-96 h post-ablation, hpa) and processed for proliferative cell nuclear antigen (PCNA) immunostaining (Fig. 1C). Mtz treatment led to PNCA staining in closely associated chains of cells spanning the inner nuclear layer (INL), consistent with proliferation of MG and MGPCs (Lahne et al., 2020) (Fig. 1D). Co-immunostaining assays performed at 1 day post-ablation (dpa) demonstrated colocalization of PCNA and glutamine synthetase (GS), which is a marker of MG cells (Fig. S1E). Quantification of PCNA-positive cells showed increased proliferation at 24 and 48 hpa after Mtz treatment (Fig. 1E). Tests in juvenile stage fish (∼6 week old) showed similarly robust PCNA staining in the INL after Mtz-induced RGC ablation (Fig. S1F). Unfortunately, transgene expression downregulation shortly thereafter precluded adult stage studies of RGC regeneration.

To test whether RGCs regenerate after ablation, Mtz-treated RGC:YFP-NTR2 larvae were allowed to recover until 11 dpf (168 hpa, Fig. 1F). Intravital confocal time series images were collected pre-Mtz treatment at 5 dpf (Cntr, 0 hpa), 48 hpa (7 dpf) and 168 hpa (11 dpf). Confocal *z*-stack projections show clear evidence of RGC loss and regeneration in the Mtz-treated group (Fig. 1F). Quantification of YFP volumes in confocal images of the tectum showed a ∼99% reduction of RGC axons at 48 hpa and a return to ∼30% of pre-ablation control levels at 168 hpa – i.e. 6 dpa (Fig. 1G).

Studies using the NTR/Mtz cell ablation system provided initial evidence of ‘fate-biased’ retinal regeneration, with MGPCs preferentially giving rise to the lost cell types after selective amacrine cell or cone photoreceptor ablation (D'Orazi et al., 2020; Emmerich et al., 2023b; Ng Chi Kei et al., 2017). Fate-biased regeneration has recently also been observed in light damage (LD) and *N*-methyl-D-aspartate (NMDA) excitotoxicity models (Lyu et al., 2023). To investigate MGPC fate choices after RGC ablation, ethynyl-2′-deoxyuridine (EdU) was pulsed with Mtz from 0-24 hpa (5-6 dpf) to label proliferating cells and then chased to 96 hpa (9 dpf) to assess MGPC differentiation (Fig. 1H). Immunostaining revealed that unablated controls had EdU-positive cells in the ciliary margin zone (CMZ) and the endothelial layer surrounding the lens, but rarely any EdU-positive cells in the GCL, INL or outer nuclear layer (ONL; Fig. 1I, Cntr). In contrast, Mtz-treated retinas showed prominent EdU staining in the GCL and rare EdU-positive cells in other retinal layers (Fig. 1I, +Mtz). Quantification showed a statistically significant increase of EdU staining in the GCL of Mtz-treated retinas (Fig. 1J). Together with the absence of EdU cells in other retinal layers at this time point in both Cntr and +Mtz images, these data suggest targeted RGC ablation induced a fate biased regenerative response, similar to selective amacrine and cone photoreceptor ablation paradigms (D'Orazi et al., 2020; Fraser et al., 2013; Ng Chi Kei et al., 2017).

We next assessed whether RGC loss and regeneration were correlated to changes in a visually driven behavior. Upon loss of light, zebrafish larvae exhibit a phototaxis response characterized by an initial area-restricted search (helical turning) followed by a roaming search pattern (Hageter et al., 2021, 2023; Horstick et al., 2020). Only the initial area-restricted search requires vision (Horstick et al., 2017). We therefore used this assay to test whether RGC-ablated larvae show altered initial responses to darkness onset. RGC ablation significantly impacted darkness-induced behaviors relative to controls at 48 hpa and 96 hpa, before returning to baseline levels at 6 dpa (Fig. 1K,L). The observed kinetics of RGC ablation and regeneration (e.g. YFP levels; Fig. 1F,G) therefore parallel the observed timing of lost and re-established visual performance in this assay.

### Transcriptomic analysis of RGC regeneration

**Fig. 2.**
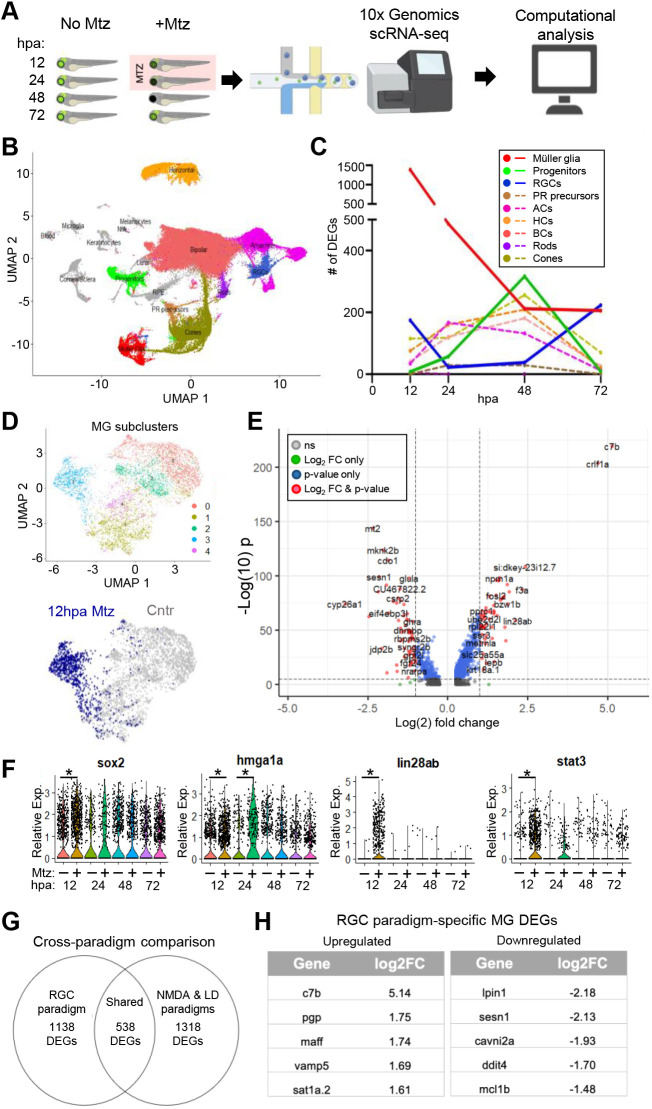
**Single cell transcriptomics of response to RGC ablation.** (A) Experimental setup for scRNA-seq time series (12, 24, 48 and 72 hpa) of unablated control (no Mtz) and RGC-ablated (+Mtz) larvae. (B) UMAP of major eye cell types from all timepoints collected*.* (C) Total number of DEGs identified at each timepoint for MG, progenitor cells, RGCs and other major retinal cell classes. (D) MG subclusters. Subcluster 3 is made up predominantly of ‘activated’ MG cells at 12 hpa (blue). (E) Volcano plot of DEGs in 12 hpa MG subcluster. (F) Select MG DEGs at 12, 24, 48 and 72 hpa. (G) Cross-paradigm comparison of MG DEGs from RGC ablation data, and a pooled NDMA and LD dataset. (H) Top upregulated and downregulated RGC paradigm-specific DEGs relative to pooled NDMA and LD dataset. ACs, amacrine cells; BCs, bipolar cells; HCs, horizontal cells; PR, photoreceptor; RGCs, retinal ganglion cells. *P≤0.05.

To gain mechanistic insights, we profiled transcriptional changes associated with RGC loss and regeneration using time resolved single-cell RNA sequencing (scRNA-seq). Whole eye samples were collected from both RGC ablated and control larvae at 12, 24, 48 and 72 hpa (Fig. 2A). A total of 133,104 cells were profiled. UMAP clustering identified all major cell classes of the eye (Fig. 2B), as confirmed by known markers (Fig. S2). A total of 4949 differentially expressed genes (DEGs) were identified (minimum FC>1.18, adjusted *P*-value <0.05, Fig. 2B, Table S1). DEGs were enriched in activated MG (1385) and dying RGCs (174) at 12 hpa, in activated MG (487) at 24 hpa, in retinal progenitor cells (MGPCs and CMZ cells; 317) at 48 hpa, and in MG (205) and RGCs (223) at 72 hpa (Fig. 2C).

To assess specific gene changes in MG after RGC ablation, the MG cluster (Fig. 2B, red) was isolated and subclustered. Five subclusters were identified, including a subcluster (designated 3) almost entirely composed of 12 hpa MG cells (Fig. 2D). A volcano plot of 12 hpa MG DEGs shows that two immune-related factors, *c7b* (Emmerich et al., 2023b; Sifuentes et al., 2016) and *crlf1a* (Laura et al., 2023), are the most highly upregulated genes in MG upon RGC ablation (Fig. 2E). Other MG activation markers enriched in 12 hpa MG included *sox2*, *hmga1a*, *lin28ab* and *stat3* (Fig. 2F). These changes are consistent with bulk RNA-seq data from purified MG after acute LD (Sifuentes et al., 2016) and/or single cell data from LD and NMDA paradigms (Hoang et al., 2020). Interestingly, *ascl1a*, a gene previously shown to be upregulated and required for MG activation in retinal tissue damage paradigms (Fausett et al., 2008; Hoang et al., 2020; Ramachandran et al., 2011), was absent from the list of DEGs enriched in activated MG after RGC ablation.

Next, we compared MG DEGs in a related dataset. Hoang et al. performed scRNA-seq of adult zebrafish retinas after widespread retinal cell loss upon LD or injection of the NMDA (Hoang et al., 2020). Pooling MG datasets from NMDA and LD paradigms identified 1856 DEGs, versus a total of 1678 MG DEGs identified in our data. Comparisons across datasets revealed 538 shared DEGs (32%), whereas 1138 were unique to the RGC paradigm (Fig. 2G, Table S2). This proportion of paradigm-specific and shared DEGs parallels a bulk RNA comparison of two selective cell ablation paradigms (Emmerich et al., 2023b). Upregulated MG DEGs unique to our dataset included *c7b*, *pgp*, *maff*, *vamp5* and *sat1a.2*, while unique downregulated genes included *lpin1*, *sesn1*, *cavin2a*, *ddit4* and *mcl1b* (Fig. 2H).

### Pseudotime trajectory identifies RGC ablation-induced genes across regeneration

**Fig. 3.**
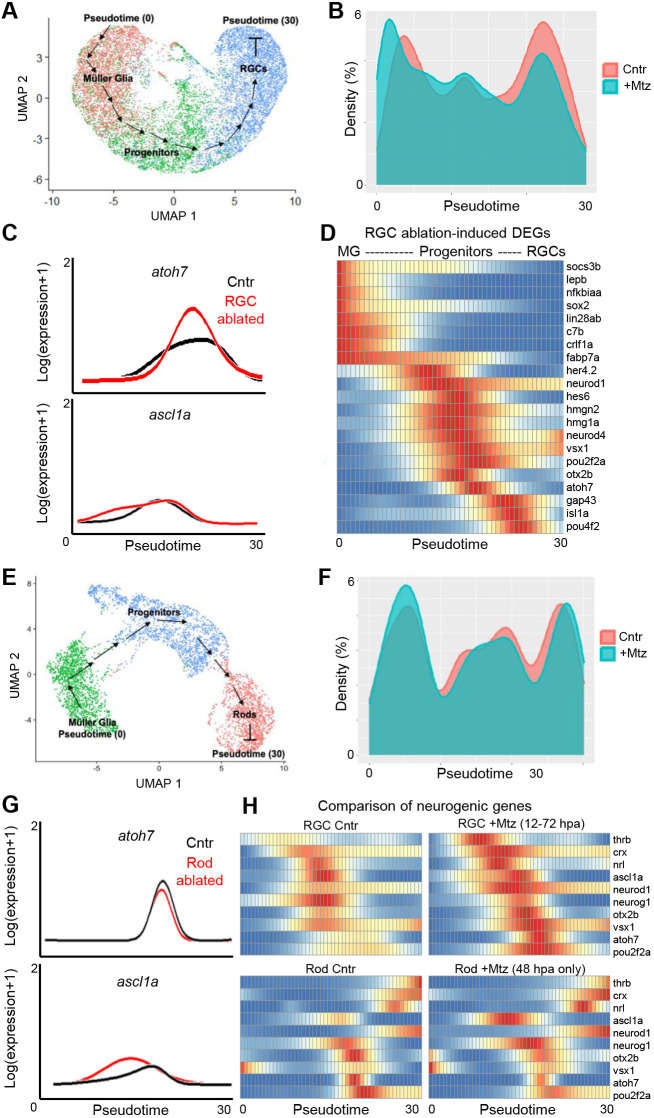
**Pseudotime trajectory analysis of RGC and rod PR regeneration paradigms.** (A) UMAP used to create pseudotime trajectory of MG>progenitor>RGC cells using 0-72 hpa data. (B) Density of unablated (Cntr) and RGC ablated (+Mtz) cells along the trajectory in A. (C) Expression of *atoh7* and *ascl1a* in Cntr (black line) and RGC-ablated data (red line) along trajectory in A. (D) Heatmap of selected DEGs upregulated during RGC loss and regeneration. (E) UMAP used to create pseudotime trajectory of MG>progenitors>rods using 48 hpa data. (F) Density of unablated (Cntr) and rod-ablated (+Mtz) cells along the trajectory in E. (G) Expression of *atoh7* and *ascl1a* in Cntr (black line) and rod-ablated (red line) data along the trajectory in E. (H) Comparison of changes in expression of selected neurogenic genes between RGC ablation (top series) or rod cell ablation (bottom series) paradigms along corresponding pseudotime trajectories.

We next employed pseudotime analyses to assess gene changes along a MG>Progenitor>RGC trajectory, akin to related studies (Hoang et al., 2020; Lyu et al., 2023; Todd et al., 2021, 2022). We isolated MG, progenitor cells (both MGPCs and CMZ cells) and RGCs from control and ablated samples, and inferred a common pseudotime trajectory (Fig. 3A). Analysis of cell density along the trajectory showed enrichment of ablated sample cells early during MG activation, less differences in the middle where progenitor cells were enriched, and enrichment for control sample cells later (Fig. 3B). 1829 DEGs were identified along pseudotime, which segregated into 11 expression patterns (Fig. S3A, Table S3). Genes upregulated in gene clusters 1 and 2 were associated with inflammation (*c7b* and *crlf1a*), MG activation (*gfap* and *lin28ab*) and DNA damage (*gadd45ab*). Genes upregulated in cluster 3 included markers of MGPCs (*pax6a*, *foxn4* and *sox2*) and RGC precursors (*atoh7* and *pou4f2*). Cluster 4-7 genes were upregulated progressively later in the trajectory, including neurogenic factors (*thrb*, *neurod1*, *neurod4*, *neurod6b* and *otx2b*) and genes associated with neuronal differentiation (*gap43* and *alcamb*). Cluster 8-11 genes were downregulated progressively later and included neurogenic factor (*zfhx3*) and mature neuronal (*elavl4*) markers. Gene ontology analysis on each cluster identified multiple developmentally important signaling pathways, including MAP kinase, JAK/STAT, Wnt, BMP and Hedgehog (Fig. S3B).

We were particularly interested in expression patterns of transcription factors (TFs) *atoh7* and *ascl1a*. During development, *atoh7* specifies the RGC lineage (Kay et al., 2001). As mentioned above, *ascl1a* has been shown to be required for retinal tissue regeneration (Fausett et al., 2008). After RGC ablation, *atoh7* was strongly induced in the progenitor phase, while *ascl1a* exhibited a minor increase earlier during the MG activation phase (Fig. 3C). A subset of the most significantly induced genes along the trajectory (Fig. 3D) included factors associated with: (1) MG activation (*lepb* and *nfkbiaa*), (2) MGPCs (*sox2*, *fabp7a* and *her4.2*), (3) neurogenesis (*neurod1*, *neurod4*, *hmgn2* and *vsx1*), (4) specification of RGCs (*atoh7*) and other early developmental cell fates (*pou2f2a*), and, lastly, (5) RGC differentiation and maturation (*isl1*, *pou4f2* and *gap43*) (Wu et al., 2015).

To assess context specificity of the RGC regeneration transcriptomic signature, we performed scRNA-seq using a published NTR/Mtz-based rod photoreceptor ablation model (Walker et al., 2012). 5 dpf larvae were treated with or without Mtz for 24 h and whole-eye samples collected at 48 hpa. Pseudotime analysis was used to construct a MG>progenitor>rod trajectory (Fig. 3E,F). In contrast to the RGC paradigm, rod ablation led to a decrease in *atoh7*, and a stronger increase in *ascl1a* expression (Fig. 3G). Comparing neurogenic gene profiles showed obvious differences related to neuronal cell type of each trajectory (Fig. 3H, RGC Cntr versus Rod Cntr), and paradigm-specific changes after induction of cell loss. RGC ablation induced relatively higher levels of *atoh7*, *pou2f2a*, *thrb* and *otx2b*, whereas rod loss led to higher relative levels of *ascl1a* and *neurog1* (Fig. 3H).

### Reverse genetics ‘crispant’ screen: knockout of *ascl1a* enhances RGC regeneration

**Fig. 4.**
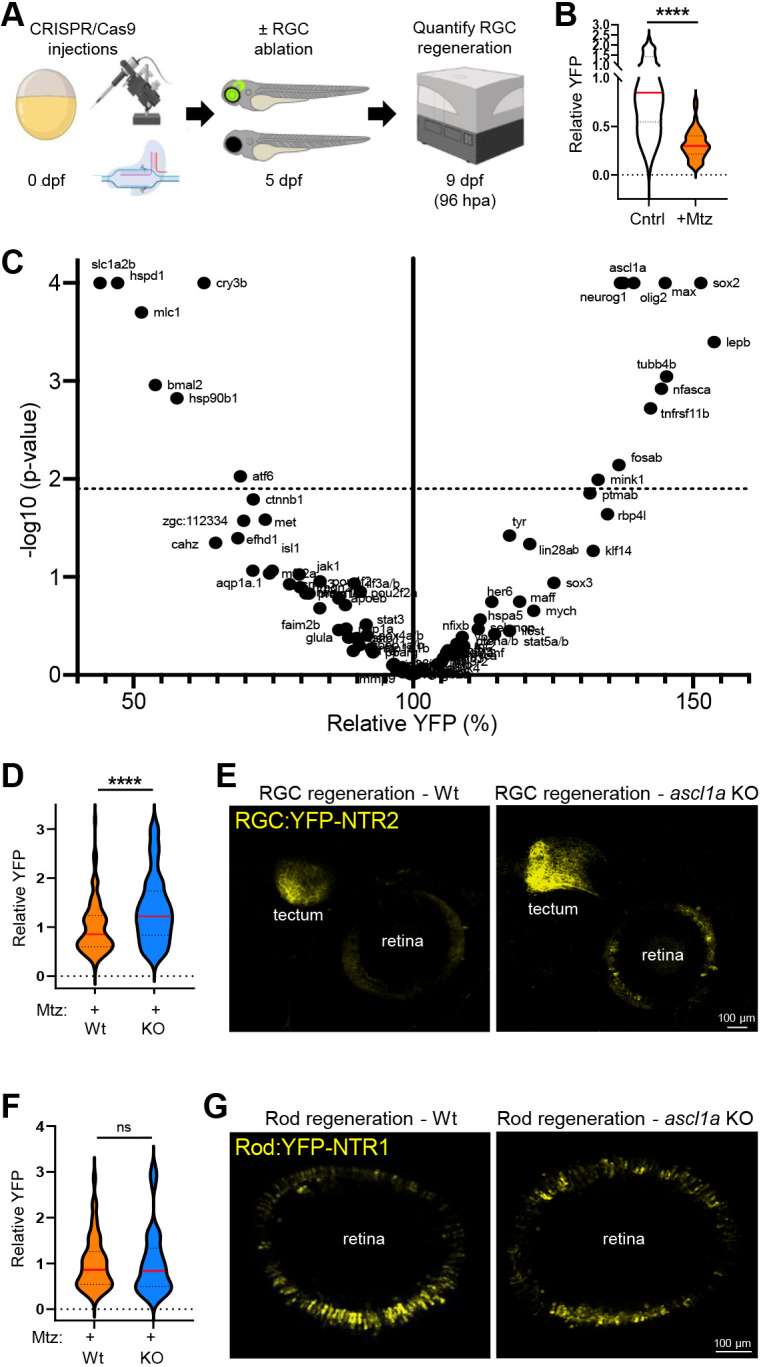
**CRISPR screen identifies genes regulating RGC regeneration.** (A) Experimental setup for CRISPR/Cas9-based ‘crispant’ knockout (KO) screen for genes that regulate RGC regeneration. (B) Plate reader-based quantification of RGC regeneration at 96 hpa. Relative YFP levels in RGC ablated larvae (+Mtz) are normalized to unablated controls (Cntr)*.* (C) Volcano plot of RGC regeneration (relative YFP levels) for all Mtz-treated RGC:YFP-NTR2 crispant KO larvae (dashed line indicates adjusted *P*-value cutoff of 0.01; minimum sample size *n*=8, median sample size *n*=16). (D) Plate reader-based quantitative comparison of RGC regeneration levels (YFP) between Mtz-treated control (Wt) and *ascl1a* crispant (KO) larvae at 96 hpa. (E) Representative confocal images of RGC regeneration in Mtz-treated control (Wt) and *ascl1a* crispant (KO) larvae at 168 hpa. (F) Plate reader-based quantitative comparison of rod cell regeneration levels (YFP) between Mtz-treated control (Wt) and *ascl1a* crispant (KO) larvae at 96 hpa. (G) Representative confocal images of rod cell regeneration in Mtz-treated control (Wt) and *ascl1a* crispant (KO) larvae at 168 hpa. ****P≤0.0001; ns, not statistically significant.

To identify regulators of RGC regeneration, we performed a large-scale reverse genetics screen. An efficient CRISPR/Cas9-based method (Wu et al., 2018) was used to create biallelic mutations in 100 genes of interest. All genes were targeted individually or as pairs (for paralogs). Briefly, fertilized RGC:YFP-NTR2 eggs were co-injected with Cas9 and four gRNAs per targeted gene. This approach induces widespread somatic mutation of the targeted loci, enabling phenotypic screens with the injected ‘crispant’ fish (Shaw and Mokalled, 2021). Crispant larvae and controls were treated with 100 μM Mtz from 5 to 6 dpf. Plate reader-based quantification of YFP was then used to screen for effects on RGC regeneration kinetics at 9 dpf (4 dpa), a time point where ∼32% of RGCs had regenerated in control wild-type larvae (Fig. 4A,B; plate reader assays quantify both cellular and axonal YFP content, thus the difference in RGC kinetics relative to confocal imaging of RGC axons only, Fig. 1F,G). Sixty-two candidate genes were chosen from our scRNA-seq dataset. Another 39 genes were selected from the literature (Hoang et al., 2020) based on being previously shown to be either required for retinal regeneration after widespread retinal damage (19 genes) or strongly implicated as being required in that process (20 genes; Tables S4 and S5). Ten crispant KOs produced developmental defects that precluded testing effects on RGC regeneration (three from the known and/or implicated set *– hmga1a*, *yap1* and *tgif1* – and seven from the scRNA-seq set – *baxa*, *pax2a/b*, *ccn2a*, *mcf2a*, *smarca5*, *xbp1* and *bmp2b*; Table S4). Most viable crispants were similar to controls, with 73 out of 91 candidates having no significant effect on RGC regeneration kinetics (Fig. 4C, Table S4; statistical significance was defined as a FDR adjusted *P*-value of ≤0.01). Among these, 28 came from the 39 genes previously known and/or implicated in retinal tissue regeneration and another 45 came from the 62 genes selected from our scRNA-seq dataset (Fig. 4C, Table S4).

Of the remaining 18 genes, 11 crispants exhibited enhanced RGC replacement kinetics (*ascl1a*, *neurog1*, *max*, *sox2*, *olig2*, *lepb*, *tubb4b*, *nfasca*, *tnfrsf11b*, *fosab* and *mink1*) while KO of seven others inhibited RGC regeneration (*slc1a2b*, *hspd1*, *cry3b*, *mlc1*, *bmal2*, *hsp90b1* and *atf6*; Fig. 4C, Table S4). However, five additional KOs trended toward increased regenerative kinetics (*ptmab*, *rbp4l*, *tyr*, *lin28ab* and *sp6*), and nine others trended toward inhibition of RGC regeneration (*ctnnb1*, *zgc:112334*, *met*, *cahz*, *efhd1*, *isl1a*, *aqp1a*.*1*, *mcf2a*, *jak1*; Fig. 4C, Table S4; trending defined as an FDR adjusted *P*-value of >0.01 to 0.1). Including the trending factors, 32 genes were implicated as regulators of RGC regeneration: 16 pro-regenerative factors (seven significant and nine trending) and 16 anti-regenerative factors (11 significant and five trending; predicted gene functions are the opposite of KO effects, thus there is a reversal of effect here). Of these, 11 had been previously implicated in retinal regeneration and 21 had not (Table S4).

Interestingly, in comparing the effects of known/implicated regulators on retinal tissue regeneration, we noted that nearly all of them exhibited discordant effects on RGC regeneration: either having no effect (28 genes) or opposite effects (7 genes) (Tables S4 and S5). For example, KO of *mmp9* had no effect on RGC regeneration, despite having roles in promoting INL and GCL fates in LD and NMDA paradigms, respectively (Table S5; Lyu et al., 2023). Similarly, *sox2* KOs showed pro-regenerative effects in the RGC paradigm but produced anti-regenerative effects in the context of LD, and overexpression led to increased proliferation in uninjured control retinas (Gorsuch et al., 2017). Additional genes where disruption led to accelerated RGC regeneration kinetics included *ascl1a* (Fausett et al., 2008), *olig2* (Fimbel et al., 2007) and *lepb* (Zhao et al., 2014). Even among the 19 genes previously shown to be ‘required’ for retinal regeneration in the context of tissue damage paradigms, 13 had no effect, and two KOs promoted (KO of *lin28* also trended toward a pro-regenerative effect) and one KO inhibited (three caused developmental defects that precluded testing; Fig. 4C, Tables S4 and S5) RGC regeneration. The only gene exhibiting a concordant effect across paradigms was *hspd1*, where KO caused anti-regenerative effects in RGC and LD paradigms (Qin et al., 2009). In all, 35 out of 36 known and/or implicated regulators of retinal regeneration after widespread tissue damage (LD, NMDA or puncture wound) had either no or opposing effects on RGC regeneration (Tables S4 and S5).

We were particularly intrigued that KO of *ascl1a* led to accelerated RGC regeneration. This gene was initially selected as our control for inhibiting RGC replacement, due to the widespread notion that it is ‘required’ for retinal regeneration (Fausett et al., 2008). Moreover, forced expression of Ascl1 in mouse MG stimulates a nascent regenerative response in the injured mouse retina (Todd et al., 2021, 2022). Follow -up plate reader assays (Fig. 4D) as well as *in vivo* imaging (Fig. 4E) confirmed primary screen results, showing that KO of *ascl1a* enhanced RGC regeneration. Additional controls showed CRISPR/Cas9-based KO of *ascl1a* was highly efficient, and had no effect on RGC development or on Mtz-induced RGC death (Fig. S4). To assess specificity, we tested the effect of *ascl1a* KO on rod photoreceptor regeneration kinetics and saw no change by either plate reader assay (Fig. 4F) or intravital imaging (Fig. 4G).

### Ascl1a knockout biases towards early progenitor cell fates in MGPCs

**Fig. 5.**
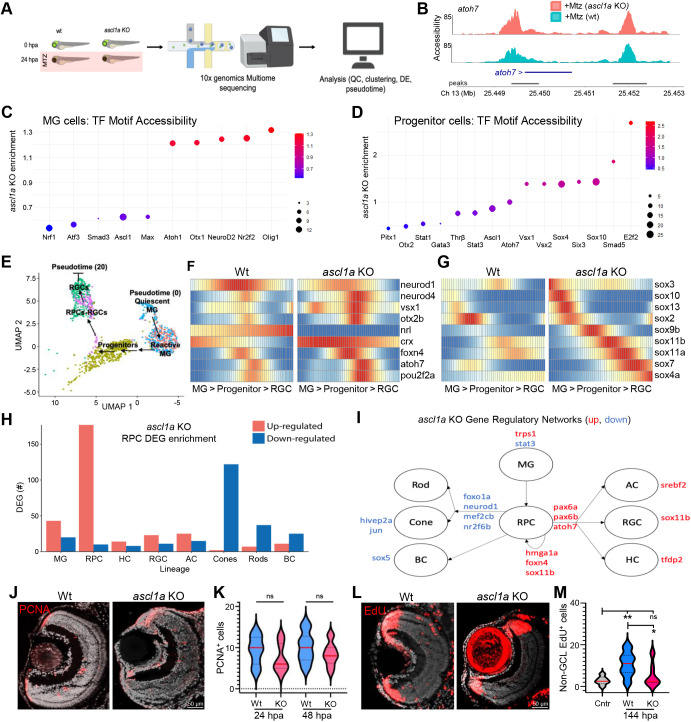
**Ascl1a KO accelerates RGC regeneration kinetics by enhancing RGC fate bias.** (A) Experimental setup for multiome sequencing (combined snRNA-seq and ATAC-seq) of control (wt) and *ascl1a* KO larvae at 0 and 24 hpa. (B) Chromatin accessibility comparison between wt and *ascl1a* KO larvae at the *atoh7* locus after RGC ablation (+Mtz). (C) Transcription factor motif accessibility enrichment in MG cells of *ascl1a* KO larvae. (D) Transcription factor motif accessibility enrichment in progenitor cells of *ascl1a* KO larvae. (E) UMAP used to create pseudotime trajectory of MG>progenitor>RGC cells. (F,G) Heatmaps of select pro-neural (F) and Sox family (G) TF DEGs across pseudotime for wild-type and *ascl1a* KO larvae at 24 hpa. (H) DEGs in progenitor cells of *ascl1a* KO larvae associated with known retinal cell lineages. (I) Derived gene regulatory networks (GRNs) altered in *ascl1a* KO progenitors based on changes in expression of transcription factors known to regulate DEGs in H. (J) Representative PCNA immunostaining in RGC-ablated control (+Mtz) and RGC-ablated *ascl1a* crispant (+Mtz, *ascl1a* KO) retinas. (K) Quantification of PCNA^+^ cells after RGC ablation at 24 and 48 hpa in wild-type and *ascl1a* KO retinas. (L) Representative EdU immunostaining images of ‘secondary’ EdU lineage-traced cells (0-48 h EdU pulse, 6-day chase) in RGC-ablated wild-type and *ascl1a* KO retinas. (M) Quantification of the EdU^+^ retinal cells in the ONL and INL (excluding CMZ and GCL) in unablated controls and RGC-ablated wild-type and *ascl1a* KO retinas at 6 dpa. *P≤0.05, **P≤0.01.

To examine how *ascl1a* KO enhanced RGC regeneration, we performed a multiomic snRNA-seq/ATAC-seq analysis on wild-type and *ascl1a* KO retinas at 0 and 24 hpa (Fig. 5A), profiling a total of 53,889 cells. UMAP clustering of an integrated dataset identified all major retinal cell classes (Fig. S5). Analysis of differential chromatin accessibility in retinal progenitor cells identified significantly increased accessibility peaks for *atoh7* in *ascl1a* KO cells at 24 hpa (Fig. 5B). We next analyzed scATAC-seq data in MG and progenitor clusters for differentially accessible transcription factor-binding motifs. Motifs with increased accessibility in *ascl1a* KO MG at 24 hpa included Atoh1, Olig1, Nr2f2, Neurod2 and Otx1, while decreased motifs included Nrf1 and Ascl1 (Fig. 5C). For *ascl1a* KO progenitor cells, motifs with increased accessibility included E2f2, Six3, Sox4/10 and Vsx1/2. Motifs with decreased accessibility included Pitx1, Otx2, Thrb, Stat1 and Ascl1 (Fig. 5D).

Next, we produced a MG>progenitor>RGC pseudotime trajectory with annotation of new subclusters made possible by chromatin data (Fig. 5E), identifying 269 significantly differentially regulated factors (Table S6). As above, we compared relative levels of neurogenic (as well as Sox family) TFs between wild-type and *ascl1a* KO retinas. Most neurogenic genes were co-expressed in progenitors, suggesting induction of a ‘transitional’ progenitor cell state (Lyu and Mu, 2021; Wu et al., 2021). Neurogenic TFs upregulated in *ascl1a* KO progenitor cells included *crx*, *foxn4*, *neurod4*, *vsx1*, *otx2b*, *pou2f2a* and *atoh7* (Fig. 5F), and all Sox gene family members evaluated, except *sox2* (Fig. 5G). Strong induction of *sox11a*, *sox11b* and *sox4a* (Fig. 5G) – both of which are implicated in RGC development in mice (Jiang et al., 2013) – further supports enhanced RGC fate bias in *ascl1a* KOs. Conversely, *neurod1* and *nrl* showed reduced levels of expression in *ascl1* KOs (Fig. 5F), suggesting reduced production of photoreceptors (Mears et al., 2001; Ochocinska and Hitchcock, 2009). The increased expression of *crx* in *ascl1a* KO MGPCs suggests they exit the cell cycle early (Muranishi et al., 2011) and/or increase cone photoreceptor differentiation (due to the downregulation of *nrl*). Both of these possibilities are consistent with MGPCs shifting towards early neuronal fates in the absence of *ascl1a*.

To further explore potential changes in cell fate, we examined changes in established lineage-promoting genes between wild-type and *ascl1a* KO progenitors. The data showed *ascl1a* KO increased the relative numbers of genes associated with RGC, horizontal cell and amacrine cell lineages, and decreased the number of genes associated with rod, cone and bipolar cell fates (Fig. 5H). To construct GRNs, we identified TFs predicted to underlie changes in *ascl1a* KO progenitor cells. TFs associated with downregulation of photoreceptor lineage genes included *neurod1*, *nr2f6b*, *foxo1a* and *mef2cb*. TFs associated with upregulation of RGC, horizontal cell and amacrine cell lineage genes included *pax6a*, *pax6b* and *atoh7* (Fig. 5I, Table S7).

Last, to investigate whether disrupting *ascl1a* altered proliferation and/or cell fate bias during RGC regeneration, we used PCNA immunolabeling and EdU lineage tracing. Interestingly, *ascl1a* KO retinas exhibited no change in PCNA cell numbers relative to wild-type controls (Fig. 5J,K). We next assessed MGPC production of cells other than RGCs as a measure of relative fate bias. Similar to a NTR-based amacrine cell regeneration paradigm, acute ablation of nearly all RGCs is predicted to subsequently cause low levels of cell loss in the INL and ONL (Ng Chi Kei et al., 2017). To track MGPCs responding to secondary cell loss, the EdU pulse was extended by 24 h (0-48 hpa) and chased until 7 dpa (12 dpf). The data showed that wild-type MGPCs gave rise to a small but statistically significant number of cells in INL and ONL after RGC ablation (Fig. 5L,M). In contrast, no significant differences were observed between unablated controls and *ascl1a* KO retinas, consistent with reduced levels of non-RGC production (Fig. 5L,M). Combined with our GRN analysis, these results suggest the mechanism by which *ascl1a* KO accelerated RGC replacement kinetics was by enhancing the level of RGC fate bias exhibited by MGPCs.

## DISCUSSION

Our results demonstrate plasticity in how MG convert to a stem cell-like state and context specificity in how genes function to regulate retinal regeneration, i.e. *ascl1a*-independent mechanisms of MG dedifferentiation and a preponderance of paradigm-specific gene effects, respectively. These findings echo emerging evidence of divergence in how retinal regeneration is regulated across paradigms, suggesting regenerative processes are informed by and adapt to the nature of the injury incurred (Emmerich et al., 2023b; Lyu et al., 2023). Where this information originates (dying and/or surviving cells), how it is transmitted, and how it controls MGPC proliferation rates and cell fate decisions are largely unresolved.

Combining a novel transgenic line enabling targeted RGC ablation with single cell multiomics and large-scale reverse genetic screening, we identified 18 genes whose disruption altered RGC regeneration kinetics. Of the seven crispants that inhibited RGC replacement, most have no known role in regeneration, except *hspd1* (Qin et al., 2009) (above) and *hsp90b1* (i.e. effects on senescence during muscle regeneration; He et al., 2019). These genes are intriguing as their normal function is predicted to promote RGC regeneration. KO of *mlc1*, a target of Notch/Rbpj signaling, may promote MG dedifferentiation to promote regeneration. Three other pro-RGC regeneration genes are involved in mediating the unfolded protein response (UPR), *hspd1*, *atf6* and *hsp90b1*, and two involved in mediating circadian rhythmicity, *bmal2* and *cry3b*. These results suggest reduction of UPR-mediated cell stress may promote regeneration in general, as this pathway has also been implicated in hair cell and tail regeneration (Lin et al., 2021; Pei et al., 2018), and add to earlier evidence of circadian gene involvement in retinal regeneration (Qin et al., 2009).

Among the 11 crispants that accelerated RGC regeneration kinetics, i.e. anti-RGC regeneration factors, five of the targeted genes are transcription factors, three are basic helix-loop-helix (bHLH) proneural factors (*ascl1a*, *olig2* and *neurog1*), one is a SRY-box factor that regulates the expression of proneural genes such as *neurog2* and *neurod1* (*sox2*; Amador-Arjona et al., 2015) and one is a bHLH predicted to regulate the cell cycle (*max*; Jones, 2004). In addition, *ascl1a*, *sox2*, *olig2* and *lepb*, stood out as genes previously shown and/or implicated as being required for retinal regeneration in the context of widespread damage (Fausett et al., 2008; Fimbel et al., 2007; Gorsuch et al., 2017; Zhao et al., 2014). How disrupting *lepb* effects RGC regeneration is less clear, as it is highly upregulated across tissue- and cellular-level regenerative paradigms (Kang et al., 2016; Zhao et al., 2014), including the RGC regeneration model presented here. In the context of a retinal puncture wound, morpholino knockdown of the *lepb* (leptin) receptor (*lepr*) decreased proliferation of cells in the INL, but also reduced expression of *ascl1a*, which provides a potential connection between *lepb* and *ascl1a* KO results here. Our most intriguing result, however, was that disruption of *ascl1a* increased RGC regeneration kinetics.

We expected *ascl1a* KO to inhibit RGC regeneration kinetics due to *ascl1a* being required for MG proliferation after puncture wounding, ouabain toxicity or LD in zebrafish (Fausett et al., 2008; Ramachandran et al., 2011) (Table S4). In addition, forced expression of Ascl1 in mouse MG cells awakens latent regenerative potential in NMDA-injected (Todd et al., 2021, 2022) and LD (Pavlou et al., 2024) retinas. Recent studies have shown that co-expressing Ascl1 with other neurogenic factors – Atoh1 (Todd et al., 2021) or Pou4f2, and Islet1 (Todd et al., 2022) – in mouse MG leads to the production of RGC-like cells that fail to mature fully. Here, loss of *ascl1a* enhanced RGC fate bias during regeneration, which is in keeping with Ascl1-expressing RPCs rarely producing RGCs during development (Brzezinski et al., 2011; VandenBosch et al., 2020). Removing *ascl1a* from the pool of neurogenic factors co-expressed in ‘transitional’ progenitor cells (Lyu and Mu, 2021; Wu et al., 2021) may therefore increase RGC differentiation probability by decreasing non-RGC options. The *ascl1a* KO scATAC-seq data support this explanation, showing increased accessibility for: (1) TFs associated with RGC production and/or early retinal cell fates, such as *atoh7* (Kay et al., 2001), *neurod2* (Cherry et al., 2011) and *sox4* (Chang et al., 2017; Jiang et al., 2013); (2) TFs whose overexpression promotes RGC-like production in mice, such as *atoh1* (Todd et al., 2021, 2022); and (3) known retinal stem/progenitor cell markers, such as *otx1* (Diacou et al., 2022), *six3* (Raven et al., 2018), *e2f2* (Dagnino et al., 1997) and *nr2f2* (Tang et al., 2010). Similarly, pseudotime data show upregulation of additional TFs that drive RGC fate, including *sox11a* and *sox11b* (Chang and Hertz, 2017; Chang et al., 2017). Increased fate bias could also account for the pro-RGC regenerative effects of knocking out two other bHLH transcription factors: *olig2* and *neurog1*. Olig2-expressing mouse retinal progenitors, similar to Ascl1-expressing progenitors, rarely produce RGCs (Hafler et al., 2012). Similarly, loss of Neurog1 increases early-born fates in the mouse cortex (Han et al., 2018), suggesting *neurog1* crispants may similarly increase RGCs as they are one of the earliest born neurons in the retina. The effect of *sox2* disruption may be dose dependent, with low levels of expression promoting precocious RGC differentiation via upregulation of *atoh7* (formerly *ath5*) and inhibition of Notch (Taranova et al., 2006). Constitutive expression of Ascl1 in mouse MG cells may actively repress RGC differentiation, thus Ascl1-independent means of dedifferentiating MG and/or methods for downregulating Ascl1 after MG dedifferentiation may be required to promote RGC maturation. Regarding Ascl1-independent dedifferentiation, several other neurogenic factors, including *sox2*, *sox10*, *neurod1* and *neurog2*, are able to reprogram astroglia cells into neurons (Talifu et al., 2022), as per overexpression of Ascl1 in mouse MG cells.

In a recent comparison of LD and NMDA excitotoxicity in zebrafish, Lyu et al., made several key observations that further support the hypothesis that the nature of the injury informs retinal regenerative processes and that underscore key differences between retinal development and retinal regeneration (Lyu et al., 2023). These insights explain why MGPC responses to LD and NMDA injury paradigms have been previously characterized as ‘multipotent’ (Powell et al., 2016), rather than responsive to injury specifics: a higher degree of cell death specificity had been assumed. Moreover, the data from Lyu et al., further support the idea that MGPCs can exhibit fate bias, clarifying that even when cell death is widespread throughout all layers of the retina, MGPCs are exquisitely attuned to the nature of the injury. In further support of this concept, single cell transcriptomics analyses revealed divergent gene expression across retinal regeneration paradigms; ∼70% of MG DEGs were paradigm specific rather than shared across RGC ablation and LD and/or NMDA models. Similarly, a comparison of two selective retinal cell ablation models, targeting either rod photoreceptor or retinal bipolar cells, showed that paradigm-specific gene changes predominated in bulk RNA datasets (Emmerich et al., 2023b).

In contrast to these findings, a recent report has shown that the type of injury incurred, either LD or NMDA, does not alter cell fate in the context of Ascl1-overexpressing mouse MG cells (Pavlou et al., 2024). Interestingly, when Atoh1 and Ascl1 are co-expressed in mouse MG, the type of injury incurred does have a small effect on the types of cells generated – 80% RGC-like versus 90% RGC-like in LD versus NMDA, respectively. Given the findings of Lyu et al., that neither LD nor NMDA-based damage paradigms induce layer-specific cell losses in zebrafish, it will be prudent to re-evaluate the specificity of cell death across all current paradigms by evaluating cell loss acutely and in subsequent days to account for secondary ‘bystander’ cell death. A simple explanation for the seeming discrepancy between the findings of Pavlou et al. and ‘fate biased’ regenerative responses observed in zebrafish is that they are due to technical differences. That is, the effects of forced expression of proneural transcription factors in mouse MG may not reflect the natural course of regeneration in zebrafish. Our interpretation of the *ascl1a* KO zebrafish data is, in fact, not inconsistent with forced Ascl1a and/or Ascl1a-Atoh1 expression having relatively invariant effects on the fate of MGPCs in mice – i.e. we would predict that this would bias MGPC toward fates specified by the overexpressed factor(s).

To test for context-specific gene function during retinal regeneration more comprehensively, 36 previously identified and/or implicated regulators of retinal regeneration in the context of widespread tissue damage were screened for effects on regeneration after selective RGC ablation. The majority of tested factors, 16 genes previously shown to be required for and 20 genes implicated in retinal tissue regeneration, had either no statistically significant effect (28 genes) or accelerated RGC regeneration kinetics (seven genes). Only one crispant showed concordant effects across paradigms, *hspd1* KO inhibiting regeneration after LD (Qin et al., 2009) and RGC ablation. In addition, a gene we previously implicated in retinal bipolar cell regeneration, *pparg*, had no effect on either rod photoreceptor (Emmerich et al., 2023b) or RGC regeneration. We also saw no effects on RGC regeneration upon targeting JAK/STAT signaling (*stat3*, *stat5a*, *stat5b* and *jak1*), which differs from results in retinal damage (Elsaeidi et al., 2014; Nelson et al., 2012; Todd et al., 2016; Zhang et al., 2005) and selective rod cell ablation models (Emmerich et al., 2023b). These results highlight the need for functional gene testing across different regenerative models to account for paradigm-specific effects.

Our lab and others have recently shown that the immune system plays a crucial role in regulating retinal regeneration (Emmerich et al., 2023a; Lyu et al., 2023; Nagashima and Hitchcock, 2021; White et al., 2017). This is consistent with immune-related factors being among the most highly upregulated genes in MG during initial phases of the response to retinal injury (Laura et al., 2023; Sifuentes et al., 2016). In addition, several immune signaling associated factors were among the genes whose disruption altered RGC regeneration kinetics, including *hspd1*, *hsp90bl* and *atf6* (among inhibitory crispants), and *lepb* and *tnfsrf11b* (among accelerated crispants). Similarly, we recently found that *c7b* crispants exhibit accelerated rod photoreceptor regeneration kinetics (Emmerich et al., 2023b). Studies in which IL6 family member expression was modulated, including the alternative CNTF receptor ligand gene *crlf1a*, demonstrate that immune signaling also controls optic nerve regeneration in zebrafish; RGC axon regrowth after optic nerve crush is inhibited in *crlf1a* morphants (Elsaeidi et al., 2014). However, here, KO of *c7b* or *crlf1a* had no effect on RGC regeneration (Table S4). These results suggest immune-mediated regulation of retinal regeneration is also context specific, with immune-related genes having potentially discordant role(s) across regeneration paradigms.

Possible explanations for the prevalence of paradigm-specific gene effects we observed are differences in age (larvae versus adults) and/or methods: gene disruption (crispants versus morphants), retinal injury (cell ablation versus LD/NMDA/puncture) and assay measure (cell replacement kinetics versus proliferation). Further experiments will be required to investigate the impact of these differences. Crispants robustly recapitulate stable mutant phenotypes (Wu et al., 2018), enabling large-scale screens of regeneration-associated genes in larvae (Keatinge et al., 2021) or adults (Shaw and Mokalled, 2021). Most earlier tests, however, involved injecting morpholinos into the eye and electroporation (Thummel et al., 2008, 2011). Phenotypic discordance between morphant and crispant tests may therefore arise due to differential immune responses, off-target effects and/or crispant mutant mRNA triggered genetic compensation (El-Brolosy et al., 2019). Genetic compensation could explain the degree of plasticity we observe and should be investigated from both an experimental and therapeutic perspective, i.e. leveraged to promote regeneration. Finally, by assessing regeneration kinetics, we were able to show that retinal cell regeneration can be promoted independently of increases in MG/MGPC proliferation, likely explaining at least some of the discordance between our findings and previous reports focused largely on effects on MG/MGPC proliferation.

In summary, our findings suggest that context-specific regulatory mechanisms govern each phase of the retinal regenerative process. However, mechanisms for reprogramming MG to a stem-like state and regulating MGPC proliferation and differentiation also appear to be inherently plastic, which has profound implications for regenerative therapeutics. Recent observations that MGPC proliferation rates are matched to the level of cell loss (Lyu et al., 2023; Wilson et al., 2016) support the use of regenerative paradigms for defining feedback mechanisms of proliferative control (Lander et al., 2009). Similarly, that fate bias may be a generalizable feature of regenerative processes (Lyu et al., 2023) – rather than a feature unique to selective cell ablation paradigms – opens new opportunities to enhance understanding of how retinal cell differentiation is controlled. Insights on these fronts could support strategies for selectively stimulating the regeneration of disease-relevant retinal neuron types.

## MATERIALS AND METHODS

### Zebrafish husbandry and transgenic lines

All studies were carried out in accordance with recommendations by the Office of Laboratory Animal Welfare (OLAW) for zebrafish studies and an approved Johns Hopkins University Animal Care and Use Committee animal protocol. All fish were maintained using established conditions at 28.5°C with a 14:10 h light:dark cycle. All larvae used were given 1-phenyl 2-thiourea (PTU) beginning at 1 dpf to facilitate screening for transgenic expression before experiments.

### Transgenic lines

The Rod:YFP-NTR1 ablation line [*Tg(rho:YFP Eco. NfsB)gmc500*] has been previously published (Emmerich et al., 2023a,b; Zhang et al., 2021). To enable bipartite transgenic targeting of RGCs (RGC:YFP-NTR2), we combined the previously published *Tg(isl2b.3:Gal4)zc65* known to label ∼95% of RGCs when combined with UAS driver elements (Fujimoto et al., 2011), with the NTR2.0 variant recently published: *Tg(5xUAS:GAP-tagYFP-P2A-nfsB_Vv F70A/F108Y)jh513* (Sharrock et al., 2022). For all experiments, larvae were screened at 5 dpf for strong YFP expression in expected regions and reduced off target expression. In the RGC:YFP-NTR2 fish, we occasionally observed off-target expression in the trigeminal neuroglia, fish with this expression pattern were not used in any quantitative assays.

### Metronidazole mediated cell ablation

Cell ablation mediated by metronidazole (Mtz) was performed by first anesthetizing 5 dpf RGC:YFP-NTR2 or Rod:YFP-NTR1 larvae with 1×MS-222 (tricaine) and screening for strong YFP expression in expected regions. Fish were then split into appropriate groups with or without Mtz and placed into six-well plates at the proper Mtz concentration diluted in 5ml of E3/PTU media. Fresh 10 mM Mtz stocks were made for each experiment.

### Plate reader-based fluorescence quantification assays

To measure relative levels of RGC development (at 5 dpf), RGC loss (at 7 dpf) and RGC or rod cell regeneration (at 9 dpf), established plate reader-based fluorescence quantification assays were performed to compare unablated and/or wild-type controls to experimental conditions using the Automated Reporter Quantification *in vivo* (ARQiv) system, as previously described (White et al., 2017).

To determine the minimum sample size required to detect hit genes in the reverse genetics screen, the standard deviation of plate reader measured YFP signals readings from 96 wild-type RGC-ablated larvae was calculated at 96 hpa (the endpoint for the screen). We then used a power calculator (ClinCalc available online based on a published technique; Rosner, 2011) to determine whether a minimum sample size of eight would enable identification of a 30% effect size on regeneration in crispant fish (alpha of 0.05, power of 80%).

### *In vivo* confocal microscopy to assess cell loss and regeneration

All intravital imaging applied previously detailed protocols (White and Mumm, 2013). Confocal *z*-stacks encompassing all retinal/brain fluorescence in the sample (step size, 5 μm) were collected. Image analysis was performed using FiJi for basic image processing (i.e. ImageJ v1.49b; NIH) or Imaris (v7.6.5; Bitplane) for nuclei counting after creation of a surface for DAPI or volumetric quantification using local background-based volumetric rendering of YFP signals.

### Statistical analyses

Statistical tests for all quantification data were carried out using GraphPad Prism 9. When more than two groups were compared, we performed one-way ANOVA tests with Dunnett's multiple comparisons correction and significance was considered an adjusted *P*-value of <0.05. For the CRISPR/Cas9 mutagenesis screen, we employed a one-way ANOVA followed by the recommended two-stage false discovery rate correction of Benjamini, Krieger, and Yekutieli, which cutoff significance at an adjusted *P*-value of 0.01.

### Tissue preparation, immunohistochemistry and confocal microscopy

For immunohistochemistry, larval zebrafish were euthanized in 20×MS-222, fixed in 4% paraformaldehyde (PFA) for at least 4 h, washed three times in 1×PBS (phosphate-buffered saline; EMD Millipore) and placed in 30% sucrose for ∼1 h. Samples were then mounted in cryogel embedding medium, frozen in liquid nitrogen then stored at −80⁰C until sectioned in the lateral plane at 10-14 µm with a cryostat. Sliced sections were collected on standard microscope slides. For immunolabeling, slides were air dried at room temperature for ∼1 h, rinsed in 1×PBS and then re-fixed with 4% PFA for 15 min. PBST rinses (1×PBS +0.1% Tween20, Fisher Scientific) were conducted to remove trace PFA followed by a 5 min antigen retrieval with SDS (1% sodium dodecyl sulfate; Fisher Scientific) in PBS. The blocking phase was performed with 3% goat serum in PBDT (1×PBS, 1% BSA, 1% DMSO and 0.1% TritonX-100) for 30 min and incubated with primary antibody/1% goat serum/PBDT overnight at 4°C. The next morning, slides were rinsed in PBST, stained with secondary antibody/PBDT for ∼2 h in a light protected humidity chamber and cover-slipped (22×50 mm, Fisher Scientific). PBST rinses removed unbound secondary antibody. Samples were protected with Vectashield+DAPI (Vector Laboratories) and cover-slipped (24×50 mm, Fisher Scientific).

For EdU lineage tracing experiments, larvae treated with or without Mtz were exposed to 80 μM EdU (Thermo Fisher) for either 24 h or 48 h and collected at indicated timepoints afterwards. Following standard sectioning and PBS washing after sectioning as above, an EdU detecting reaction was performing according to the manufacturer's instructions (Click-iT EdU Cell Proliferation Kit for Imaging, Alexa. Fluor 647 dye, Thermo Fisher). After EdU reaction steps, slides that received additional antibodies received primary antibody staining as above. EdU^+^ vascular endothelial cells were delineated by their presence immediately interiorly to the ganglion cell layer and their distinctive elongated nuclei, a well-established morphological hallmark of these cells (Hedberg-Buenz et al., 2016).

Primary antibodies included: rabbit anti-PCNA monoclonal antibody (1:500; Millipore Sigma, SAB2701819, Lot GT40541), mouse anti-glutamine synthetase (1:200; Millipore Sigma, MAB302, Lot LV1501431) and mouse anti-HuC/D (Elavl3/4; 1:500; Invitrogen, A21272, Lot 2566335). Secondary antibodies included: goat anti-mouse Alexa Fluor 594 (1:500; Life Technologies, A11032, Lot 2527968) and goat anti-rabbit Alexa Fluor 430 (1:500; Life Technologies, A11064, Lot 1310273). Immunostaining was performed as previously described (White et al., 2017).

Images were collected with an Olympus FV1000 confocal microscope (405, 440, 488, 515, 559 and 635 nm laser lines). Stacked confocal images were obtained using a 40×oil immersion objective with a 5 µm step size, 130 µm aperture and 10 µm total depth. Five or six sections were collected per retina centered around the optic nerve. Images (Olympus .oib format) were analyzed using ImageJ. Manual cell counts were for PCNA and EdU^+^ cells were averaged for each group.

### CRISPR/Cas9 mediated targeting

CRISPR/Cas9-mediated redundant targeting injections were carried out using the published strategy and gRNA table of Wu et al. (2018). Four oligos per target were ordered as DNA oligos, assembled with the general CRISPR tracr oligo, and then transcribed using pooled *in vitro* transcription (HiScribe T7 High Yield RNA Synthesis kit, New England BioLabs) and cleaned up with the NEB Monarch RNA Cleanup kit. A mixture of all four sgRNAs per targeted gene (1 ng in total) and Cas9 protein (2.5 µM, IDT) were injected into one-cell stage embryos. When paralogs of the targeted gene were present, both paralogs were targeted by injecting four sgRNAs per paralog; e.g. a total of eight sgRNAs when targeting two paralogs. For each injection cycle, gRNAs for the tyrosinase gene were injected that allow the confirmation of injection efficacy by looking for reduced pigmentation in injected embryos at 2 dpf.

### PCR and qPCR to verify *ascl1a* knockout

After *ascl1a* 4xgRNA injections, validation of DNA cutting was performed by DNA extraction, PCR and 1% gel electrophoresis with the following primers (forward, CCGCGAACACGTTCCCAATGGA; reverse, TGACACTCGGGACCCGTGGTTT) using the genotyping method in the GeneWeld paper (Welker et al., 2021). To validate that mRNA expression was reduced after knockout, 2 dpf embryos were processed for quantitative PCR using a published protocol (Emmerich et al., 2023b). Briefly, extracted mRNA samples were reverse transcribed (Qiagen Omniscript RT kit, Qiagen) and stored at −20°C. Samples were run in triplicate using the BioRad iQ SYBR Green Supermix (BioRad) in iCycler IQ 96-well PCR plates (Bio-Rad) on a BioRad iCycler equipped with an iCycler iQ Detection System. The protocol consisted of three phases: (1) 95°C for 10 min, (2) 50 cycles at 95°C for 15 s and 60°C for 1 min, and a final melt curve analysis ramp from 55°C to 95°C at 0.5°C per step (5 s per step). β-Actin served as the house-keeping gene and the 2^−ΔΔCT^ method was used for normalization to ensure equal amounts of cDNA for comparisons. qPCR primers were designed using the online tool QuantPrime.

### Single cell sample preparation and RNA sequencing

For each sample eyes were collected from 20-30 larval fish (40-60 retinas) in sibling unablated or post-injury transgenic fish (12/24/48/72 hpa for RGC:YFP-NTR2 and 48 hpa in Rod:YFP-NTR1). Retinal cells were processed through published protocols for 10x genomics as previously described (Hoang et al., 2020). Library preparation was then performed according to 10x genomics protocols for the version 3.1 kit and sequencing was conducted through the Johns Hopkins Single & Transcriptomics Core on a NovaSeq at ∼500 million reads per library.

### Single-cell RNA sequencing analysis

Raw reads were mapped to the *Danio rerio* GRCz10 using Cell Ranger vX.X from 10x genomics. Aligned genomic reads were then read into the published Seurat pipeline (v4.3.0.1) and quality control was performed by removing any cells with <200 detected genes or 1000 UMIs, and genes detected in fewer than three cells per experiment. Clustering steps were performed using steps from the pbmc Seurat tutorial available online. Briefly, the top 2000 variable genes were identified and used to identify principal components (PCs) of the data. The top 30 PCs were used to produce a UMAP and clusters were annotated with known zebrafish marker genes (Hoang et al., 2020). Differentially expressed genes (DEGs) were identified using the FindAllMarkers function between each control and ablation timepoint in each retinal cell cluster (minimum log2 foldchange cutoff of 0.25). Volcano plots showing log_2_ fold change and –log_10_ *P*-values of DEGs identified in 12 hpa MG cells were generated in R with the EnhancedVolcano package.

### Pseudotime trajectory analyses

Slingshot (v2.4.0) was used to infer pseudotime trajectories after subsetting of datasets into clusters of interest (i.e. MG, RPCs and appropriate neurons depending on the dataset). MG were treated as the root cluster and then the getLineages and getCurves functions were used to produce trajectories. In each case, common trajectories were learned using two conditions – either ablated and control cells, or wild-type and crispant cells from each appropriate experiment. The slingPseudotime function was used to calculate pseudotime for each cell and measure expression of each gene in pseudotime bins. Last, tradeSeq (v1.10.0) was used to compare gene expression between conditions (e.g. at pseudotime bin 1- calculate expression in control and ablated cells), yielding lists of differentially expressed factors.

### Single cell multiome sample preparation and sequencing

For each sample, eyes were collected from 20-30 larval fish (40-60 retinas) and flash frozen in dry ice for ∼15 min before being transferred to a −80°C freezer for storage. Nuclei were extracted from frozen retinal tissues according to the 10xMultiome ATAC+Gene Expression (GEX) protocol (CGOOO338). Briefly, frozen retinal tissues were lysed in ice-cold 500 ml of 0.1×Lysis buffer using a pestle and incubated on ice for 6 min in total. Nuclei were centrifuged, washed three times and resuspended in 10xMultiome nuclei buffer at a concentration of ∼3000-5000 nuclei/ml. Nuclei (∼15 K) then were loaded onto 10x Genomic Chromium Controller, with a target number of ∼10 K nuclei per sample. RNA and ATAC libraries were prepared according to the 10xMultiome ATAC+Gene Expression protocol, and subjected for Illumina NovaSeq sequencing at ∼500 million reads per library.

### Single-cell multiome sequencing analysis

RNA expression data were processed as above. Peak calling from single nuclei ATAC-seq reads was performed using MACS2 in the ArchR package (v1.0.2). ATAC-seq data were then processed using the pbmc scATAC-seq workflow with the Signac (v1.10.0) and Seurat (v4.3.0.1) packages for quality control, normalization and producing an integrated UMAP. Differential expression and accessibility was then calculated for both gene RNA expression and chromatin peak accessibility. Next, the ChromVar package (v1.18.0) was used to identify differentially accessible transcription factor motifs between wild-type and *ascl1a* crispant cells.

### Identification of marker genes and differentially expressed genes for gene regulatory networks

To identify the differentially expressed genes (DEGs) in the RPC cell group between the control and ascl1a KO 24 hpa injury samples, we employed the ‘findMarkers’ function from the Seurat (v4.3.0.1) package. DEGs were defined using the criteria: logfc.threshold>0.2, min.pct=0.05 and *P*-value<0.05. Marker genes for each neuronal cell type were identified using the ‘findAllMarkers’ function in Seurat with the following parameters: adjusted *P*-value<0.05, min.pct=0.05 and logfc>0.5.

### Gene regulatory network construction

Using the snRNA-seq data from control and injury samples, we inferred TF-target relationships using the Arboreto package (v0.1.6) (Aibar et al., 2017) in Python. We obtained importance scores for each TF-target pair using the ‘grnboost2’ function. We then filtered the TF-target pairs based on these importance scores, removing pairs with scores lower than the 95th quantile. Additionally, we calculated the Pearson correlation for each TF-target pair based on the cell-by-gene expression matrix. A TF-target pair was annotated as ‘positive’ regulation if its correlation (cor) was>0.03 and as ‘negative’ regulation if its correlation (cor) was<−0.03. Any other TF-target relationships were discarded. Finally, we filtered the GRNs based on TF expression in RPCs. Any TF not expressed in RPCs was filtered out.

### Identifying neuron-biased TFs upon *ascl1a* knockout

From the DEGs identified in RPCs of *ascl1a* knockouts, we noted that gene expression changes in *ascl1a* knockout RPCs favor MG, RPCs, HC, RGC and AC fates, and diminish cone, rod and BC fates. To delve deeper into the TFs correlated with these gene expression biases, we identified RPC-expressed proneural TFs associated with specific neuronal fates [similar to a recent study (Lyu et al., 2023)]. With derived GRNs, we determined a cell-type specificity score (*P*-value) for each TF using a hypergeometric test in R (‘phyper’ function).

## Supplementary Material



10.1242/develop.202754_sup1 Supplementary information

Table S1. DEGs associated with RGC loss and regeneration - single-cell RNA sequencing time course. Time-resolved differentially expressed genes (DEGs) identified following RGC ablation (12, 24, 48, and 72 hpa) delineated per each major retinal cell type.

Table S2. Comparison of MG DEGs identified in RGC versus widespread retinal injury paradigms. All MG DEGs associated either uniquely or shared between our RGC ablation and regeneration single cell dataset and a combined NMDA and LD single cell dataset (Hoang et al., 2020).

Table S3. Pseudotime DEGs identified in RGC regeneration trajectory. Following identification of MG, progenitor cells, and RGCs, we developed a common pseudotime trajectory (segmented into 50 timepoints) from MG to progenitor cells to RGCs (MG>Progenitors>RGCs) in unablated (Cntr) and RGC ablated (+Mtz) samples. A total of 1,829 DEGs were identified along this trajectory. Each row contains the gene, the maximum change in expression (delta expression = normalized ablated expression - unablated expression) and the gene ontology cluster term each gene belongs to.

Table S4. Summary of CRISPR screen results. For all genes tested in the large-scale crispant screen, we show the effect on RGC regeneration kinetics (percent change relative to wt controls), adjusted FDR p-value. Genes are separated as having a known (K), implicated (I), or unknown (U) role in retinal regeneration, as well as those having deleterious effects on development that precluded testing (nd).

Table S5. Comparison of effects of genes known to regulate regeneration across tested paradigms. The specific effect(s) of "known" genes previously shown to have a role in regulating retinal regeneration in the context of a puncture wound (poke), optic nerve crush (ON crush), light damage (LD), NMDA, ouabain toxicity and/or RGC ablation are compared between the tested paradigms. The change in expression (<, >, no change), assay(s) performed, test (KD, KO, OE), effect(s) on proliferation, neurite outgrowth, and/or reporter detection, effect on regeneration, and reference links are provided.

Table S6. Pseudotime DEGs identified in RGC regeneration trajectory in *ascl1a* KO. Relative expression changes of 269 significant DEGs identified along pseudotime trajectory from MG>Progenitors>RGCs comparing 24 hpa *ascl1a* KO and wt retinas processed for multiome sequencing. Relative expression is compared across 50 bins of pseudotime where >0 indicates increased expression in *ascl1a* KO retinas (max of 1) and values <0 indicated increased expression in wt retinas.

Table S7. TFs underlying GRNs in *ascl1a* KO progenitor cells. Lineage-associated progenitor cell DEGs were identified by comparing 24 hpa multiome datasets of control (wt) and *ascl1a* KO samples. Transcription factors (TFs) known to regulate these DEGs were identified and then significantly enriched TFs between conditions were analyzed. Then, gene regulatory networks (GRNs) were derived from the enriched TFs between conditions, using the Arboreto, based on previously established relationships of TFs to regulation of retinal cell type lineages in development.

## References

[DEV202754C1] Aibar, S., González-Blas, C. B., Moerman, T., Huynh-Thu, V. A., Imrichova, H., Hulselmans, G., Rambow, F., Marine, J.-C., Geurts, P., Aerts, J. et al. (2017). SCENIC: single-cell regulatory network inference and clustering. *Nat. Methods* 14, 1083-1086. 10.1038/nmeth.4463 28991892 PMC5937676

[DEV202754C2] Amador-Arjona, A., Cimadamore, F., Huang, C.-T., Wright, R., Lewis, S., Gage, F. H. and Terskikh, A. V. (2015). SOX2 primes the epigenetic landscape in neural precursors enabling proper gene activation during hippocampal neurogenesis. *Proc. Natl. Acad. Sci. USA* 112, E1936-E1945. 10.1073/pnas.1421480112 25825708 PMC4403144

[DEV202754C3] Bernardos, R. L., Barthel, L. K., Meyers, J. R. and Raymond, P. A. (2007). Late-stage neuronal progenitors in the retina are radial Müller Glia that function as retinal stem cells. *J. Neurosci.* 27, 7028-7040. 10.1523/JNEUROSCI.1624-07.2007 17596452 PMC6672216

[DEV202754C4] Brzezinski, J. A., Kim, E. J., Johnson, J. E. and Reh, T. A. (2011). Ascl1 expression defines a subpopulation of lineage-restricted progenitors in the mammalian retina. *Development* 138, 3519-3531. 10.1242/dev.064006 21771810 PMC3143566

[DEV202754C5] Chang, K.-C. and Hertz, J. (2017). SoxC transcription factors in retinal development and regeneration. *Neural Regen. Res.* 12, 1048-1051. 10.4103/1673-5374.211178 28852381 PMC5558478

[DEV202754C6] Chang, K.-C., Hertz, J., Zhang, X., Jin, X.-L., Shaw, P., Derosa, B. A., Li, J. Y., Venugopalan, P., Valenzuela, D. A., Patel, R. D. et al. (2017). Novel regulatory mechanisms for the SoxC transcriptional network required for visual pathway development. *J. Neurosci.* 37, 4967-4981. 10.1523/JNEUROSCI.3430-13.2017 28411269 PMC5426184

[DEV202754C7] Cherry, T. J., Wang, S., Bormuth, I., Schwab, M., Olson, J. and Cepko, C. L. (2011). NeuroD factors regulate cell fate and neurite stratification in the developing retina. *J. Neurosci.* 31, 7365-7379. 10.1523/JNEUROSCI.2555-10.2011 21593321 PMC3135085

[DEV202754C8] Dagnino, L., Fry, C. J., Bartley, S. M., Farnham, P., Gallie, B. L. and Phillips, R. A. (1997). Expression patterns of the E2F family of transcription factors during mouse nervous system development. *Mech. Dev.* 66, 13-25. 10.1016/S0925-4773(97)00083-X 9376316

[DEV202754C9] Diacou, R., Nandigrami, P., Fiser, A., Liu, W., Ashery-Padan, R. and Cvekl, A. (2022). Cell fate decisions, transcription factors and signaling during early retinal development. *Prog. Retin. Eye Res.* 91, 101093. 10.1016/j.preteyeres.2022.101093 35817658 PMC9669153

[DEV202754C10] D'Orazi, F. D., Suzuki, S. C., Darling, N., Wong, R. O. and Yoshimatsu, T. (2020). Conditional and biased regeneration of cone photoreceptor types in the zebrafish retina. *J. Comp. Neurol.* 528, 2816-2830. 10.1002/cne.24933 32342988 PMC8496684

[DEV202754C11] El-Brolosy, M. A., Kontarakis, Z., Rossi, A., Kuenne, C., Günther, S., Fukuda, N., Kikhi, K., Boezio, G. L. M., Takacs, C., Lai, S.-L. et al. (2019). Genetic compensation triggered by mutant mRNA degradation. *Nature* 568, 193-197. 10.1038/s41586-019-1064-z 30944477 PMC6707827

[DEV202754C12] Elsaeidi, F., Bemben, M. A., Zhao, X.-F. and Goldman, D. (2014). Jak/Stat signaling stimulates zebrafish optic nerve regeneration and overcomes the inhibitory actions of Socs3 and Sfpq. *J. Neurosci.* 34, 2632-2644. 10.1523/JNEUROSCI.3898-13.2014 24523552 PMC3921430

[DEV202754C13] Emmerich, K., White, D. T., Kambhampati, S. P., Casado, G. L., Fu, T.-M., Chunawala, Z., Sahoo, A., Nimmagadda, S., Krishnan, N., Saxena, M. T. et al. (2023a). Nanoparticle-based targeting of microglia improves the neural regeneration enhancing effects of immunosuppression in the zebrafish retina. *Commun. Biol.* 6, 534. 10.1038/s42003-023-04898-9 37202450 PMC10193316

[DEV202754C14] Emmerich, K., Walker, S. L., Wang, G., White, D. T., Ceisel, A., Wang, F., Teng, Y., Chunawala, Z., Graziano, G., Nimmagadda, S. et al. (2023b). Transcriptomic comparison of two selective retinal cell ablation paradigms in zebrafish reveals shared and cell-specific regenerative responses. *PLoS Genet.* 19, e1010905. 10.1371/journal.pgen.1010905 37819938 PMC10593236

[DEV202754C15] Fausett, B. V., Gumerson, J. D. and Goldman, D. (2008). The proneural basic helix-loop-helix gene ascl1a is required for retina regeneration. *J. Neurosci.* 28, 1109-1117. 10.1523/JNEUROSCI.4853-07.2008 18234889 PMC2800945

[DEV202754C16] Fimbel, S. M., Montgomery, J. E., Burket, C. T. and Hyde, D. R. (2007). Regeneration of inner retinal neurons after intravitreal injection of ouabain in zebrafish. *J. Neurosci.* 27, 1712-1724. 10.1523/JNEUROSCI.5317-06.2007 17301179 PMC6673754

[DEV202754C17] Fraser, B., DuVal, M. G., Wang, H. and Allison, W. T. (2013). Regeneration of cone photoreceptors when cell ablation is primarily restricted to a particular cone subtype. *PLoS One* 8, e55410. 10.1371/journal.pone.0055410 23383182 PMC3559598

[DEV202754C18] Fujimoto, E., Gaynes, B., Brimley, C. J., Chien, C.-B. and Bonkowsky, J. L. (2011). Gal80 intersectional regulation of cell-type specific expression in vertebrates. *Dev. Dyn.* 240, 2324-2334. 10.1002/dvdy.22734 21905164 PMC3178006

[DEV202754C19] Goldman, D. (2014). Müller glia cell reprogramming and retina regeneration. *Nat. Rev. Neurosci.* 15, 431-442. 10.1038/nrn3723 24894585 PMC4249724

[DEV202754C20] Gorsuch, R. A., Lahne, M., Yarka, C. E., Petravick, M. E., Li, J. and Hyde, D. R. (2017). Sox2 regulates Müller glia reprogramming and proliferation in the regenerating zebrafish retina via Lin28 and Ascl1a. *Exp. Eye Res.* 161, 174-192. 10.1016/j.exer.2017.05.012 28577895 PMC5554723

[DEV202754C21] Hafler, B. P., Surzenko, N., Beier, K. T., Punzo, C., Trimarchi, J. M., Kong, J. H. and Cepko, C. L. (2012). Transcription factor Olig2 defines subpopulations of retinal progenitor cells biased toward specific cell fates. *Proc. Natl. Acad. Sci. USA* 109, 7882-7887. 10.1073/pnas.1203138109 22543161 PMC3356608

[DEV202754C22] Hageter, J., Waalkes, M., Starkey, J., Copeland, H., Price, H., Bays, L., Showman, C., Laverty, S., Bergeron, S. A. and Horstick, E. J. (2021). Environmental and molecular modulation of motor individuality in larval zebrafish. *Front. Behav. Neurosci.* 15, 777778. 10.3389/fnbeh.2021.777778 34938167 PMC8685292

[DEV202754C23] Hageter, J., Starkey, J. and Horstick, E. J. (2023). Thalamic regulation of a visual critical period and motor behavior. *Cell Rep.* 42, 112287. 10.1016/j.celrep.2023.112287 36952349 PMC10514242

[DEV202754C24] Han, S., Dennis, D. J., Balakrishnan, A., Dixit, R., Britz, O., Zinyk, D., Touahri, Y., Olender, T., Brand, M., Guillemot, F. et al. (2018). A non-canonical role for the proneural gene Neurog1 as a negative regulator of neocortical neurogenesis. *Development* 145, dev157719. 10.1242/dev.157719 30201687 PMC6198467

[DEV202754C25] He, M. Y., Xu, S. B., Qu, Z. H., Guo, Y. M., Liu, X. C., Cong, X. X., Wang, J. F., Low, B. C., Li, L., Wu, Q. et al. (2019). Hsp90β interacts with MDM2 to suppress p53-dependent senescence during skeletal muscle regeneration. *Aging Cell* 18, e13003. 10.1111/acel.13003 31313490 PMC6718578

[DEV202754C26] Hedberg-Buenz, A., Christopher, M. A., Lewis, C. J., Fernandes, K. A., Dutca, L. M., Wang, K., Scheetz, T. E., Abramoff, M. D., Libby, R. T., Garvin, M. K. et al. (2016). Quantitative measurement of retinal ganglion cell populations via histology-based random forest classification. *Exp. Eye Res.* 146, 370-385. 10.1016/j.exer.2015.09.011 26474494 PMC4841761

[DEV202754C27] Hoang, T., Wang, J., Boyd, P., Wang, F., Santiago, C., Jiang, L., Yoo, S., Lahne, M., Todd, L. J., Jia, M. et al. (2020). Gene regulatory networks controlling vertebrate retinal regeneration. *Science* 370, eabb8598. 10.1126/science.abb8598 33004674 PMC7899183

[DEV202754C28] Horstick, E. J., Bayleyen, Y., Sinclair, J. L. and Burgess, H. A. (2017). Search strategy is regulated by somatostatin signaling and deep brain photoreceptors in zebrafish. *BMC Biol.* 15, 4. 10.1186/s12915-016-0346-2 28122559 PMC5267475

[DEV202754C29] Horstick, E. J., Bayleyen, Y. and Burgess, H. A. (2020). Molecular and cellular determinants of motor asymmetry in zebrafish. *Nat. Commun.* 11, 1170. 10.1038/s41467-020-14965-y 32127541 PMC7054361

[DEV202754C30] Jiang, Y., Ding, Q., Xie, X., Libby, R. T., Lefebvre, V. and Gan, L. (2013). Transcription factors SOX4 and SOX11 function redundantly to regulate the development of mouse retinal ganglion cells. *J. Biol. Chem.* 288, 18429-18438. 10.1074/jbc.M113.478503 23649630 PMC3689985

[DEV202754C31] Jones, S. (2004). An overview of the basic helix-loop-helix proteins. *Genome Biol.* 5, 226. 10.1186/gb-2004-5-6-226 15186484 PMC463060

[DEV202754C32] Jorstad, N. L., Wilken, M. S., Grimes, W. N., Wohl, S. G., VandenBosch, L. S., Yoshimatsu, T., Wong, R. O., Rieke, F. and Reh, T. A. (2017). Stimulation of functional neuronal regeneration from Müller glia in adult mice. *Nature* 548, 103-107. 10.1038/nature23283 28746305 PMC5991837

[DEV202754C33] Kang, J., Hu, J., Karra, R., Dickson, A. L., Tornini, V. A., Nachtrab, G., Gemberling, M., Goldman, J. A., Black, B. L. and Poss, K. D. (2016). Modulation of tissue repair by regeneration enhancer elements. *Nature* 532, 201-206. 10.1038/nature17644 27049946 PMC4844022

[DEV202754C34] Kay, J. N., Finger-Baier, K. C., Roeser, T., Staub, W. and Baier, H. (2001). Retinal ganglion cell genesis requires Lakritz, a zebrafish atonal homolog. *Neuron* 30, 725-736. 10.1016/S0896-6273(01)00312-9 11430806

[DEV202754C35] Keatinge, M., Tsarouchas, T. M., Munir, T., Porter, N. J., Larraz, J., Gianni, D., Tsai, H.-H., Becker, C. G., Lyons, D. A. and Becker, T. (2021). CRISPR gRNA phenotypic screening in zebrafish reveals pro-regenerative genes in spinal cord injury. *PLoS Genet.* 17, e1009515. 10.1371/journal.pgen.1009515 33914736 PMC8084196

[DEV202754C36] Kölsch, Y., Hahn, J., Sappington, A., Stemmer, M., Fernandes, A. M., Helmbrecht, T. O., Lele, S., Butrus, S., Laurell, E., Arnold-Ammer, I. et al. (2021). Molecular classification of zebrafish retinal ganglion cells links genes to cell types to behavior. *Neuron* 109, 645-662.e9. 10.1016/j.neuron.2020.12.003 33357413 PMC7897282

[DEV202754C37] Lahne, M., Nagashima, M., Hyde, D. R. and Hitchcock, P. F. (2020). Reprogramming Müller Glia to regenerate retinal neurons. *Annu. Rev. Vis. Sci.* 6, 171-193. 10.1146/annurev-vision-121219-081808 32343929 PMC8384111

[DEV202754C38] Lander, A. D., Gokoffski, K. K., Wan, F. Y. M., Nie, Q. and Calof, A. L. (2009). Cell lineages and the logic of proliferative control. *PLoS Biol.* 7, e1000015. 10.1371/journal.pbio.1000015 19166268 PMC2628408

[DEV202754C39] Laura, C., Fabian, R., Anja, M., Juliane, B., Andreas, D., Anke, W., Stefan, H. and Michael, B. (2023). Single cell RNA sequencing unravels the transcriptional network underlying zebrafish retina regeneration. *Elife* 12, RP86507. 10.7554/eLife.86507 37988404 PMC10662954

[DEV202754C40] Lenkowski, J. R. and Raymond, P. A. (2014). Müller glia: stem cells for generation and regeneration of retinal neurons in teleost fish. *Prog. Retin. Eye Res.* 40, 94-123. 10.1016/j.preteyeres.2013.12.007 24412518 PMC3999222

[DEV202754C41] Lin, Y.-F., Sam, J. and Evans, T. (2021). Sirt1 promotes tissue regeneration in zebrafish through regulating the mitochondrial unfolded protein response. *iScience* 24, 103118. 10.1016/j.isci.2021.103118 34622167 PMC8479786

[DEV202754C42] Lyu, J. and Mu, X. (2021). Genetic control of retinal ganglion cell genesis. *Cell. Mol. Life Sci.* 78, 4417-4433. 10.1007/s00018-021-03814-w 33782712 PMC8164989

[DEV202754C43] Lyu, P., Iribarne, M., Serjanov, D., Zhai, Y., Hoang, T., Campbell, L. J., Boyd, P., Palazzo, I., Nagashima, M., Silva, N. J. et al. (2023). Common and divergent gene regulatory networks control injury-induced and developmental neurogenesis in zebrafish retina. *Nat. Commun.* 14, 8477. 10.1038/s41467-023-44142-w 38123561 PMC10733277

[DEV202754C44] Mears, A. J., Kondo, M., Swain, P. K., Takada, Y., Bush, R. A., Saunders, T. L., Sieving, P. A. and Swaroop, A. (2001). Nrl is required for rod photoreceptor development. *Nat. Genet.* 29, 447-452. 10.1038/ng774 11694879

[DEV202754C45] Muranishi, Y., Terada, K., Inoue, T., Katoh, K., Tsujii, T., Sanuki, R., Kurokawa, D., Aizawa, S., Tamaki, Y. and Furukawa, T. (2011). An essential role for RAX homeoprotein and NOTCH–HES signaling in Otx2 expression in embryonic retinal photoreceptor cell fate determination. *J. Neurosci.* 31, 16792-16807. 10.1523/JNEUROSCI.3109-11.2011 22090505 PMC6633304

[DEV202754C46] Nagashima, M. and Hitchcock, P. F. (2021). Inflammation regulates the multi-step process of retinal regeneration in Zebrafish. *Cells* 10, 783. 10.3390/cells10040783 33916186 PMC8066466

[DEV202754C47] Nagashima, M., Barthel, L. K. and Raymond, P. A. (2013). A self-renewing division of zebrafish Müller glial cells generates neuronal progenitors that require N-cadherin to regenerate retinal neurons. *Development* 140, 4510-4521. 10.1242/dev.090738 24154521 PMC3817940

[DEV202754C48] Nelson, C. M., Gorsuch, R. A., Bailey, T. J., Ackerman, K. M., Kassen, S. C. and Hyde, D. R. (2012). Stat3 defines three populations of Müller Glia and is required for initiating maximal Müller Glia proliferation in the regenerating zebrafish retina. *J. Comp. Neurol.* 520, 4294-4311. 10.1002/cne.23213 22886421 PMC3478445

[DEV202754C49] Ng Chi Kei, J., Currie, P. D. and Jusuf, P. R. (2017). Fate bias during neural regeneration adjusts dynamically without recapitulating developmental fate progression. *Neural Dev.* 12, 12. 10.1186/s13064-017-0089-y 28705258 PMC5508679

[DEV202754C50] Ochocinska, M. J. and Hitchcock, P. F. (2009). NeuroD regulates proliferation of photoreceptor progenitors in the retina of the zebrafish. *Mech. Dev.* 126, 128-141. 10.1016/j.mod.2008.11.009 19121642 PMC2646809

[DEV202754C51] Pavlou, M., Probst, M., Blasdel, N., Prieve, A. R. and Reh, T. A. (2024). The impact of timing and injury mode on induced neurogenesis in the adult mammalian retina. *Stem Cell Rep.* 19, 239-253. 10.1016/j.stemcr.2023.12.010 PMC10874861 38278154

[DEV202754C52] Pei, W., Xu, L., Huang, S. C., Pettie, K., Idol, J., Rissone, A., Jimenez, E., Sinclair, J. W., Slevin, C., Varshney, G. K. et al. (2018). Guided genetic screen to identify genes essential in the regeneration of hair cells and other tissues. *NPJ Regen. Med.* 3, 11. 10.1038/s41536-018-0050-7 29872546 PMC5986822

[DEV202754C53] Powell, C., Cornblath, E., Elsaeidi, F., Wan, J. and Goldman, D. (2016). Zebrafish Müller glia-derived progenitors are multipotent, exhibit proliferative biases and regenerate excess neurons. *Sci. Rep.* 6, 24851. 10.1038/srep24851 27094545 PMC4837407

[DEV202754C54] Qin, Z., Barthel, L. K. and Raymond, P. A. (2009). Genetic evidence for shared mechanisms of epimorphic regeneration in zebrafish. *Proc. Natl. Acad. Sci. USA* 106, 9310-9315. 10.1073/pnas.0811186106 19474300 PMC2695073

[DEV202754C55] Ramachandran, R., Zhao, X.-F. and Goldman, D. (2011). Ascl1a/Dkk/beta-catenin signaling pathway is necessary and glycogen synthase kinase-3beta inhibition is sufficient for zebrafish retina regeneration. *Proc. Natl. Acad. Sci. USA* 108, 15858-15863. 10.1073/pnas.1107220108 21911394 PMC3179085

[DEV202754C56] Raven, D., Yilin, Z., Deyou, Z., Ales, C. and Wei, L. (2018). Six3 and Six6 are jointly required for the maintenance of multipotent retinal progenitors through both positive and negative regulation. *Cell Rep.* 25, 2510-2523.e4. 10.1016/j.celrep.2018.10.106 30485816 PMC6317371

[DEV202754C57] Rosner, B. (2011). *Fundamentals of Biostatistics*. 7th edn. Brooks/Cole.

[DEV202754C58] Sharma, P. and Ramachandran, R. (2022). Retina regeneration: lessons from vertebrates. *Oxf. Open Neurosci.* 1, kvac012. 10.1093/oons/kvac012 38596712 PMC10913848

[DEV202754C59] Sharrock, A. V., Mulligan, T. S., Hall, K. R., Williams, E. M., White, D. T., Zhang, L., Emmerich, K., Matthews, F., Nimmagadda, S., Washington, S. et al. (2022). NTR 2.0: a rationally engineered prodrug-converting enzyme with substantially enhanced efficacy for targeted cell ablation. *Nat. Methods* 19, 205-215. 10.1038/s41592-021-01364-4 35132245 PMC8851868

[DEV202754C60] Shaw, D. K. and Mokalled, M. H. (2021). Efficient CRISPR/Cas9 mutagenesis for neurobehavioral screening in adult zebrafish. *G3* 11, jkab089. 10.1093/g3journal/jkab089 33742663 PMC8496216

[DEV202754C61] Sifuentes, C. J., Kim, J.-W., Swaroop, A. and Raymond, P. A. (2016). Rapid, dynamic activation of Müller Glial stem cell responses in zebrafish. *Invest. Ophthalmol. Vis. Sci.* 57, 5148-5160. 10.1167/iovs.16-19973 27699411 PMC5054728

[DEV202754C62] Talifu, Z., Liu, J.-Y., Pan, Y.-Z., Ke, H., Zhang, C.-J., Xu, X., Gao, F., Yu, Y., Du, L.-J. and Li, J.-J. (2022). In vivo astrocyte-to-neuron reprogramming for central nervous system regeneration: a narrative review. *Neural Regen. Res.* 18, 750-755. 10.4103/1673-5374.353482 PMC9700087 36204831

[DEV202754C63] Tang, K., Xie, X., Park, J.-I., Jamrich, M., Tsai, S. and Tsai, M.-J. (2010). COUP-TFs regulate eye development by controlling factors essential for optic vesicle morphogenesis. *Development* 137, 725-734. 10.1242/dev.040568 20147377 PMC2827684

[DEV202754C64] Taranova, O. V., Magness, S. T., Fagan, B. M., Wu, Y., Surzenko, N., Hutton, S. R. and Pevny, L. H. (2006). SOX2 is a dose-dependent regulator of retinal neural progenitor competence. *Genes Dev.* 20, 1187-1202. 10.1101/gad.1407906 16651659 PMC1472477

[DEV202754C65] Thummel, R., Kassen, S. C., Montgomery, J. E., Enright, J. M. and Hyde, D. R. (2008). Inhibition of Müller glial cell division blocks regeneration of the light-damaged zebrafish retina. *Dev. Neurobiol.* 68, 392-408. 10.1002/dneu.20596 18161852 PMC3711086

[DEV202754C66] Thummel, R., Bailey, T. J. and Hyde, D. R. (2011). In vivo electroporation of morpholinos into the adult zebrafish retina. *J. Vis. Exp.* 58, e3603. 10.3791/3603 PMC3369653 22231802

[DEV202754C67] Todd, L., Squires, N., Suarez, L. and Fischer, A. J. (2016). Jak/Stat signaling regulates the proliferation and neurogenic potential of Müller glia-derived progenitor cells in the avian retina. *Sci. Rep.* 6, 35703. 10.1038/srep35703 27759082 PMC5069623

[DEV202754C68] Todd, L., Hooper, M. J., Haugan, A. K., Finkbeiner, C., Jorstad, N., Radulovich, N., Wong, C. K., Donaldson, P. C., Jenkins, W., Chen, Q. et al. (2021). Efficient stimulation of retinal regeneration from Müller glia in adult mice using combinations of proneural bHLH transcription factors. *Cell Rep.* 37, 109857. 10.1016/j.celrep.2021.109857 34686336 PMC8691131

[DEV202754C69] Todd, L., Jenkins, W., Finkbeiner, C., Hooper, M. J., Donaldson, P. C., Pavlou, M., Wohlschlegel, J., Ingram, N., Rieke, F. and Reh, T. A. et al. (2022). Reprogramming Müller glia to regenerate ganglion-like cells in adult mouse retina with developmental transcription factors. *Sci. Adv.* 8, eabq7219. 10.1126/sciadv.abq7219 36417510 PMC9683702

[DEV202754C70] Ueki, Y., Wilken, M. S., Cox, K. E., Chipman, L., Jorstad, N., Sternhagen, K., Simic, M., Ullom, K., Nakafuku, M. and Reh, T. A. (2015). Transgenic expression of the proneural transcription factor Ascl1 in Müller glia stimulates retinal regeneration in young mice. *Proc. Natl. Acad. Sci. USA* 112, 13717-13722. 10.1073/pnas.1510595112 26483457 PMC4640735

[DEV202754C71] VandenBosch, L. S., Wohl, S. G., Wilken, M. S., Hooper, M., Finkbeiner, C., Cox, K., Chipman, L. and Reh, T. A. (2020). Developmental changes in the accessible chromatin, transcriptome and Ascl1-binding correlate with the loss in Müller Glial regenerative potential. *Sci. Rep.* 10, 13615. 10.1038/s41598-020-70334-1 32788677 PMC7423883

[DEV202754C72] Walker, S. L., Ariga, J., Mathias, J. R., Coothankandaswamy, V., Xie, X., Distel, M., Köster, R. W., Parsons, M. J., Bhalla, K. N., Saxena, M. T. et al. (2012). Automated reporter quantification in vivo: high-throughput screening method for reporter-based assays in zebrafish. *PLoS One* 7, e29916. 10.1371/journal.pone.0029916 22238673 PMC3251595

[DEV202754C73] Welker, J. M., Wierson, W. A., Almeida, M. P., Mann, C. M., Torrie, M. E., Ming, Z., Ekker, S. C., Clark, K. J., Dobbs, D. L., Essner, J. J. et al. (2021). GeneWeld: efficient targeted integration directed by short homology in zebrafish. *Bio-Protoc.* 11, e4100. 10.21769/BioProtoc.4100 34395736 PMC8329467

[DEV202754C74] White, D. T. and Mumm, J. S. (2013). The nitroreductase system of inducible targeted ablation facilitates cell-specific regenerative studies in zebrafish. *Methods* 62, 232-240. 10.1016/j.ymeth.2013.03.017 23542552 PMC3723733

[DEV202754C75] White, D. T., Sengupta, S., Saxena, M. T., Xu, Q., Hanes, J., Ding, D., Ji, H. and Mumm, J. S. (2017). Immunomodulation-accelerated neuronal regeneration following selective rod photoreceptor cell ablation in the zebrafish retina. *Proc. Natl. Acad. Sci. USA* 114, E3719-E3728. 10.1073/pnas.1617721114 28416692 PMC5422825

[DEV202754C76] Wilson, S. G., Wen, W., Pillai-Kastoori, L. and Morris, A. C. (2016). Tracking the fate of her4 expressing cells in the regenerating retina using her4:Kaede zebrafish. *Exp. Eye Res.* 145, 75-87. 10.1016/j.exer.2015.11.002 26616101 PMC4842143

[DEV202754C77] Wu, F., Kaczynski, T. J., Sethuramanujam, S., Li, R., Jain, V., Slaughter, M. and Mu, X. (2015). Two transcription factors, Pou4f2 and Isl1, are sufficient to specify the retinal ganglion cell fate. *Proc. Natl. Acad. Sci. USA* 112, E1559-E1568. 10.1073/pnas.1413493112 25775587 PMC4386335

[DEV202754C78] Wu, R. S., Lam, I. I., Clay, H., Duong, D. N., Deo, R. C. and Coughlin, S. R. (2018). A rapid method for directed gene knockout for screening in G0 zebrafish. *Dev. Cell* 46, 112-125.e4. 10.1016/j.devcel.2018.06.003 29974860

[DEV202754C79] Wu, F., Bard, J. E., Kann, J., Yergeau, D., Sapkota, D., Ge, Y., Hu, Z., Wang, J., Liu, T. and Mu, X. (2021). Single cell transcriptomics reveals lineage trajectory of retinal ganglion cells in wild-type and Atoh7-null retinas. *Nat. Commun.* 12, 1465. 10.1038/s41467-021-21704-4 33674582 PMC7935890

[DEV202754C80] Zhang, S. S.-M., Liu, M.-G., Kano, A., Zhang, C., Fu, X.-Y. and Barnstable, C. J. (2005). STAT3 activation in response to growth factors or cytokines participates in retina precursor proliferation. *Exp. Eye Res.* 81, 103-115. 10.1016/j.exer.2005.01.016 15978261

[DEV202754C81] Zhang, L., Chen, C., Fu, J., Lilley, B., Berlinicke, C., Hansen, B., Ding, D., Wang, G., Wang, T., Shou, D. et al. (2021). Large-scale phenotypic drug screen identifies neuroprotectants in zebrafish and mouse models of retinitis pigmentosa. *Elife* 10, e57245. 10.7554/eLife.57245 34184634 PMC8425951

[DEV202754C82] Zhao, X.-F., Wan, J., Powell, C., Ramachandran, R., Myers, M. G. and Goldman, D. (2014). Leptin and IL-6 family cytokines synergize to stimulate Müller glia reprogramming and retina regeneration. *Cell Rep.* 9, 272-284. 10.1016/j.celrep.2014.08.047 25263554 PMC4194149

[DEV202754C83] Zhou, M., Bear, J., Roberts, P. A., Janiak, F. K., Semmelhack, J., Yoshimatsu, T. and Baden, T. (2020). Zebrafish retinal ganglion cells asymmetrically encode spectral and temporal information across visual space. *Curr. Biol.* 30, 2927-2942.e7. 10.1016/j.cub.2020.05.055 32531283 PMC7416113

