# Supplementary material for: A large-scale CRISPR screen reveals context-specific genetic regulation of retinal ganglion cell regeneration
Source: Development. 2024 Aug 12;151(15):dev202754. doi: 10.1242/dev.202754 (PMC11361637; doi:10.1242/dev.202754)
Supplement: Supplementary information [file develop-151-202754-s1.pdf]

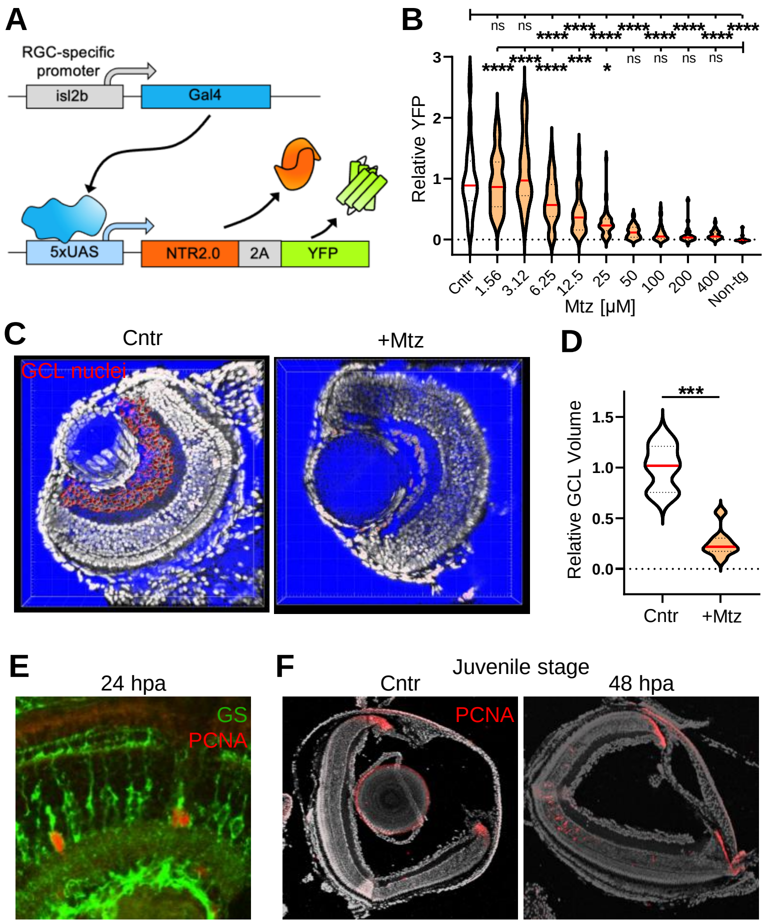

Fig. S1. Additional characterization of the RGC:YFP-NTR2 model.

(**A**) Schematic of *isl2b* promoter-based Gal4 driver and UAS reporter/effector transgenes (alleles zc65 and jh513, respectively) enabling co-expression of NTR2.0 and YFP exclusively in RGC cells. (**B**) Plate reader-based quantification of YFP in RGC:YFP-NTR2 larvae following a 48 h Mtz exposure at the indicated micromolar concentrations. (**C**) Imaris-based rendering of DAPI-stained nuclei (GCL nuclei highlighted red) in 7 dpf retinas larvae treated ±100 μM Mtz from 5-6 dpf. (**D**) Imaris-based volumetric quantification of GCL nuclei in unablated (Cntr) and RGC ablated (+Mtz) retinal sections. (**E**) Representative image of PCNA (red) and glutamine synthetase (GS, green) immunolabeled retina following Mtz-induced RGC ablation. (**F**) PCNA immunostaining in juvenile stage (6 week) RGC:YFP-NTR2 fish following Mtz-induced ablation. White violin plots are unablated (Cntr) and orange violin plots are RGC ablated (+Mtz) larvae. Asterisks in data plots indicate pair-wise (single lines) or multiple comparisons corrected (extended lines) p-value range: *≤0.05, **≤0.01, ***≤0.001, ****≤0.0001, ns=not statistically significant.

Development • Supplementary information

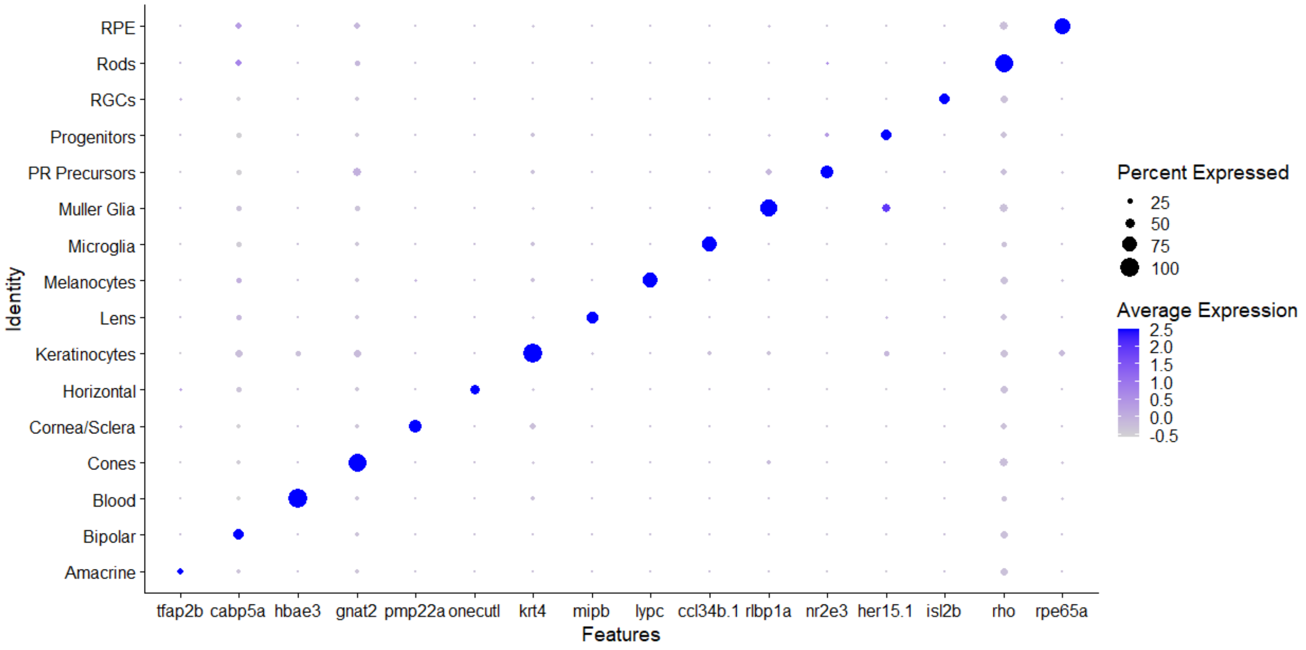

Fig. S2. Identification of major retinal cell types using established marker genes. Marker genes used to establish cell types in scRNA data.

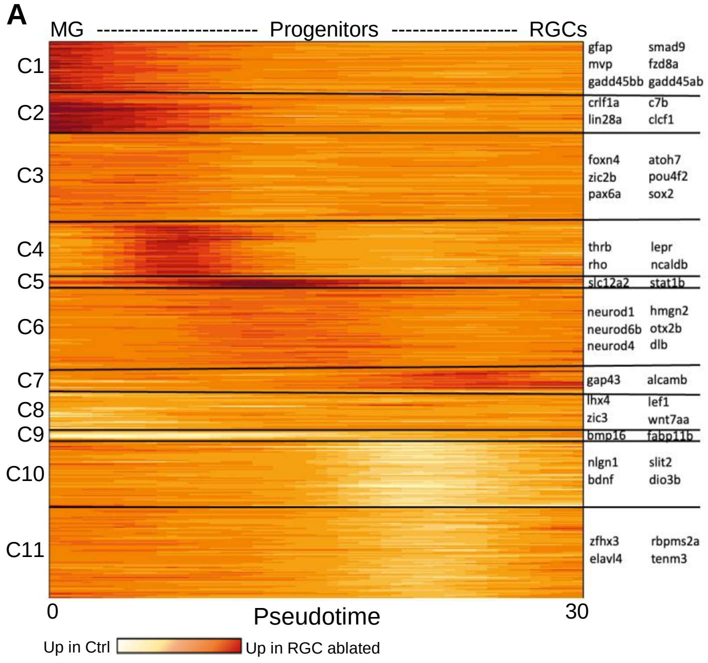

B

Cluster	GO enriched terms
C1	Positive regulation of MAP kinase, regulation of notch signaling, regulation of histone acetylation, JAK-STAT cascade
C2	Inactivation of MAPK, phosphorylation of STAT protein, positive regulation of neuron projection regeneration, regulation of canonical Wnt signaling
C3	Camera-type eye morphogenesis, regulation of retinoic acid receptor signaling, neuron development, regulation of neurogenesis
C4	Regulation of axon guidance, photoreceptor cell differentiation, positive regulation of STAT cascade
C5	Negative regulation of Wnt signaling, hemopoiesis, neuropeptide signaling
C6	Mitotic nuclear division, regulation of DNA replication, chromatin remodeling, retinal cone cell differentiation, neurogenesis
C7	BMP signaling pathway, neuron projection regeneration, Wnt signaling pathway, radial glial cell differentiation, stress activated MAPK signaling
C8	Regulation of retinal ganglion cell axon guidance, optic nerve development, eye development
C9	Retinoic acid metabolic process, regulation of BMP signaling pathway
C10	Modulation of chemical synaptic transmission, neuron cell-cell adhesion
C11	Hedgehog signaling pathway, calcium signaling pathway

Fig. S3. DEGs in clusters associated with GO terms identified in pseudotime trajectory. (**A**) Subtractive heatmap (+Mtz - Cntr samples) of 1,829 pseudotime-implicated DEGs defined by 11 Gene Ontology (GO) defined subclusters (C1-C11), example genes for each cluster shown at right. **(B)** Gene ontology terms for significantly enriched pathways associated with each cluster.

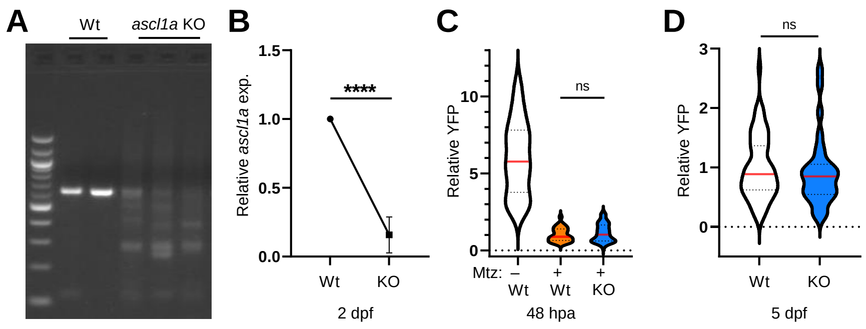

Fig. S4. Disruption of *ascl1a* had no effect on Mtz-induced cell death or RGC development. (**A**) Gel of PCR amplified fragments of *ascl1a* coding sequence from genomic DNA of wildtype (Wt) and ascl1a crispant (KO) larvae at 2 dpf. (**B**) Quantitative PCR (qPCR) based quantification of loss of *ascl1a* mRNA expression in crispant larvae (KO) normalized to wildtype (Wt) levels. (**C**) Plate reader-based quantification of YFP (RGC number) to assess effects of *ascl1a* disruption on RGC ablation efficacy comparing unablated controls (-Mtz, Wt), ablated controls (+Mtz, Wt) and ablated *ascl1a* crispants (+Mtz, KO) at 48 hpa (7 dpf). (**D**) Plate reader-based quantification of YFP (RGC number) to assess effects of *ascl1a* disruption on RGC development comparing controls (Wt) and *ascl1a* crispants (KO) at 5 dpf. Asterisks in data plots indicate pair-wise comparison p-value range: ****≤0.0001, ns=not statistically significant.

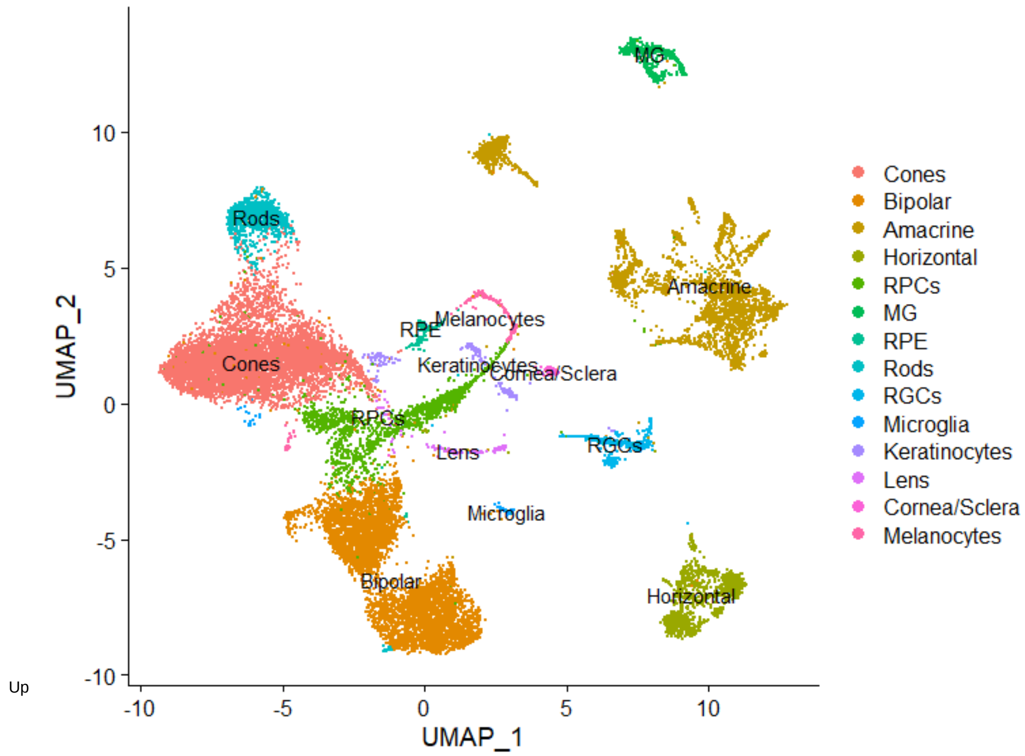

Fig. S5. Cell types identified from multiomic sequencing datasets. Identification of major eye cells types in integrated UMAP.

Development • Supplementary information

Table S1. DEGs associated with RGC loss and regeneration - single-cell RNA sequencing time course. Time-resolved differentially expressed genes (DEGs) identified following RGC ablation (12, 24, 48, and 72 hpa) delineated per each major retinal cell type.

Available for download at
https://journals.biologists.com/dev/article-lookup/doi/10.1242/dev.202754#supplementary-data

Table S2. Comparison of MG DEGs identified in RGC versus widespread retinal injury paradigms. All MG DEGs associated either uniquely or shared between our RGC ablation and regeneration single cell dataset and a combined NMDA and LD single cell dataset (Hoang et al., 2020).

Available for download at
https://journals.biologists.com/dev/article-lookup/doi/10.1242/dev.202754#supplementary-data

Table S3. Pseudotime DEGs identified in RGC regeneration trajectory.
Following identification of MG, progenitor cells, and RGCs, we developed a common pseudotime trajectory (segmented into 50 timepoints) from MG to progenitor cells to RGCs (MG>Progenitors>RGCs) in unablated (Cntr) and RGC ablated (+Mtz) samples. A total of 1,829 DEGs were identified along this trajectory. Each row contains the gene, the maximum change in expression (delta expression = normalized ablated expression – unablated expression) and the gene ontology cluster term each gene belongs to.

Available for download at
https://journals.biologists.com/dev/article-lookup/doi/10.1242/dev.202754#supplementary-data

Table S4. Summary of CRISPR screen results.
For all genes tested in the large-scale crispant screen, we show the effect on RGC regeneration kinetics (percent change relative to wt controls), adjusted FDR p-value. Genes are separated as having a known (K), implicated (I), or unknown (U) role in retinal regeneration, as well as those having deleterious effects on development that precluded testing (nd).

Available for download at
https://journals.biologists.com/dev/article-lookup/doi/10.1242/dev.202754#supplementary-data

Development • Supplementary information

Table S5. Comparison of effects of genes known to regulate regeneration across tested paradigms. The specific effect(s) of “known” genes previously shown to have a role in regulating retinal regeneration in the context of a puncture wound (poke), optic nerve crush (ON crush), light damage (LD), NMDA, ouabain toxicity and/or RGC ablation are compared between the tested paradigms. The change in expression (<, >, no change), assay(s) performed, test (KD, KO, OE), effect(s) on proliferation, neurite outgrowth, and/or reporter detection, effect on regeneration, and reference links are provided.

Available for download at
https://journals.biologists.com/dev/article-lookup/doi/10.1242/dev.202754#supplementary-data

Table S6. Pseudotime DEGs identified in RGC regeneration trajectory in *ascl1a* KO.
Relative expression changes of 269 significant DEGs identified along pseudotime trajectory from MG>Progenitors>RGCs comparing 24 hpa *ascl1a* KO and wt retinas processed for multiome sequencing. Relative expression is compared across 50 bins of pseudotime where >0 indicates increased expression in *ascl1a* KO retinas (max of 1) and values <0 indicated increased expression in wt retinas.

Available for download at
https://journals.biologists.com/dev/article-lookup/doi/10.1242/dev.202754#supplementary-data

Table S7. TFs underlying GRNs in *ascl1a* KO progenitor cells.
Lineage-associated progenitor cell DEGs were identified by comparing 24 hpa multiome datasets of control (wt) and *ascl1a* KO samples. Transcription factors (TFs) known to regulate these DEGs were identified and then significantly enriched TFs between conditions were analyzed. Then, gene regulatory networks (GRNs) were derived from the enriched TFs between conditions, using the Arboreto, based on previously established relationships of TFs to regulation of retinal cell type lineages in development.

Available for download at
https://journals.biologists.com/dev/article-lookup/doi/10.1242/dev.202754#supplementary-data

Development • Supplementary information
