# Supplementary material for: A large-scale CRISPR screen reveals context-specific genetic regulation of retinal ganglion cell regeneration
Source: Development. 2024 Aug 12;151(15):dev202754. doi: 10.1242/dev.202754 (PMC11361637; doi:10.1242/dev.202754)
Supplement: Supplementary file 1 [file develop-151-202754_review_history.pdf]

A large-scale CRISPR screen reveals context-specific genetic regulation of retinal ganglion cell regeneration

Kevin Emmerich, John Hageter, Thanh Hoang, Pin Lyu, Abigail V. Sharrock, Anneliese Ceisel, James Thierer, Zeeshaan Chunawala, Saumya Nimmagadda, Isabella Palazzo, Frazer Matthews, Liyun Zhang, David T. White, Catalina Rodriguez, Gianna Graziano, Patrick Marcos, Adam May, Tim Mulligan, Barak Reibman, Meera T. Saxena, David F. Ackerley, Jiang Qian, Seth Blackshaw, Eric Horstick and Jeff S. Mumm
DOI: 10.1242/dev.202754

Editor: Kenneth Poss

Review timeline

Original submission:	1 February 2024
Editorial decision:	3 April 2024
First revision received:	2 July 2024
Accepted:	8 July 2024

Original submission

First decision letter

MS ID#: DEVELOP/2024/202754

MS TITLE: Molecular regulation of retinal regeneration is context specific

AUTHORS: Kevin Emmerich, John Hageter, Thanh Hoang, Abigail V Sharrock, Pin Lyu, Anneliese Ceisel, James Thierer, Zeeshaan Chunawala, Saumya Nimmagadda, Isabella Palazzo, Liyun Zhang, Frazer Matthews, David T White, Catalina Rodriguez, Gianna Graziano, Patrick Marcos, Adam May, Tim Mulligan, Barak Reibman, Meera T Saxena, David F Ackerley, Jiang Qian, Seth Blackshaw, Eric Horstick, and Jeff S Mumm

Please accept my apologies for the delay in getting this decision to you. The referees' comments are appended below, or you can access them online: please go to BenchPress and click on the 'Manuscripts with Decisions' queue in the Author Area.

As you'll see, the reviewers are enthusiastic and the overall evaluation is positive. We would like to publish a revised manuscript in Development, provided that the referees' comments can be satisfactorily addressed. In looking at these I feel they are for the most part editorial requesting text edits, new discussion, modification of figures, or clarity. Please attend to all of the reviewers' comments in your revised manuscript and detail them in your point-by-point response. If you do not agree with any of their criticisms or suggestions explain clearly why this is so. If it would be helpful, you are welcome to contact us to discuss your revision in greater detail. Please send us a point-by-point response indicating your plans for addressing the referees' comments, and we will look over this and provide further guidance.

Reviewer 1

Advance summary and potential significance to field

© 2024. Published by The Company of Biologists under the terms of the Creative Commons Attribution License (https://creativecommons.org/licenses/by/4.0/).

This is an excellently executed study examining how cell type specific ablation in zebrafish retina impacts cell regeneration and compares to broader injury approaches using scRNAseq, multiomics and a substantial amount of CRISPR/Cas9 KO models for validation. The authors selectively ablated retinal ganglion cells (RGCs) using elegant transgenic cell specific reporter and ablation system. Intriguingly, the authors found that Muller Glia (MG), the active progenitor required for repair of the neural retina transcriptionally respond differently to the different injury types. The majority of differentially expressed genes in MG during retinal repair were injury type specific. One hundred candidate genes involved in MG response were selected for CRISPR/Cas9 KO and validated for regeneration retinal ganglion identifying eleven key genes that accelerate RGC regeneration and seven that inhibited RGC repair. For further study asc1a was chosen because of the surprising finding that KO ascl1a is accelerating RGC repair. Ascl1 has previously been viewed as pro-regenerative in the zebrafish retina and overexpression in mouse MG stimulate repair. To identify further mechanisms a scMultiomics approach was performed on ascl1a KO retina after injury and key regulatory mechanisms and shift in cell bias was identified explaining the accelerated generation of RGCs in ascl1a KO fish. Essentially the ascl1a KO MG becomes “primed” and shift towards the RGC lineage and accelerating fate decision towards RGC production during repair. Overall this is a fantastic study that I have no problems in recommending after minor corrections (see below).

Minor comments

Overall, some attention to text required as text in result is quite succinct.

-In abstract please reword/breakup this sentence in abstract its too complex and confusing

“Here, combining a novel zebrafish model enabling inducible selective cell ablation with single cell multiomics and CRISPR/Cas9-based knockout methods, we screened 100 candidate genes for effects on the regeneration of retinal ganglion cells (RGCs), the cells lost in glaucoma; identifying 7 genes that promote and 11 genes that inhibit RGC regeneration.”

-The connection to disease/glaucoma comes a bit abrupt and out of the context in sections/sentences below. Its important to highlight the role of cell types in disease but it really disrupt reading flow as context of text is completely different (regeneration mechanisms). Please move and edit it just doesn’t work out well as it is.

In abstract “the regeneration of retinal ganglion cells (RGCs), the cells lost in glaucoma”

Introduction “New RGCs and photoreceptors, the cells most closely associated with retinal disease(11,12), are rarely observed.”

-Comment on statement “More recently, overexpression of Ascl1 and Atoh1 resulted in mammalian MG becoming “RGC-like” cells(13), showing cell fate can be modulated to promote the regeneration”. Did this study actually show that Ascl1 or Atoh1 programmed “RGC-like” cells become RGC or function like RGC or where they just “RGC-like”?

-In Figure 2. Please increase font size in the pesudotime plots. Its very hard read text among the cells. Please also check that text is readable in other tables/plots

-Relating to CRISPR/Cas9 screen. Four guides were used to target one gene. Were injections targeting only one gene at a time or were several gene targets addressed in one injection? To me appears its one gene/injection is that correct?

-Fig S4 A. Is coding sequence referring to transcript/cDNA or genomic/exon?
-Any shared mechanisms between KO genes that improved RGC regeneration? Or the ones inhibiting RGC regeneration? Please comment/dicsuss

-What is causing the accelerated RGC regeneration in the KO fish? Could it be shorter cell cycle, pre-mature/rapid differentiation or priming/biasing MGCPs towards RGC fate? I realise by reading entire manuscript that this is well answered for the ascl1a. Is there any evidence that cells would differentiate it quicker or is it consequence of fate bias?

© 2024. Published by The Company of Biologists under the terms of the Creative Commons Attribution License (https://creativecommons.org/licenses/by/4.0/).

-The message of this sentence does come through very clearly. Please revise
"Consistent with this, functional tests of the 33 genes known/implicated in regeneration in the context of widespread damage (LD, NMDA, puncture wounds) found that most genes affected only a single retinal regenerative paradigm or had opposing effects in different models, such as ascl1a and sox2 (table S4).

Comments for the author

This is an excellently executed study examining how cell type specific ablation in zebrafish retina impacts cell regeneration and compares to broader injury approaches using scRNAseq, multiomics and a substantial amount of CRISPR/Cas9 KO models for validation. The authors selectively ablated retinal ganglion cells (RGCs) using elegant transgenic cell specific reporter and ablation system. Intriguingly, the authors found that Muller Glia (MG), the active progenitor required for repair of the neural retina transcriptionally respond differently to the different injury types. The majority of differentially expressed genes in MG during retinal repair were injury type specific. One hundred candidate genes involved in MG response were selected for CRISPR/Cas9 KO and validated for regeneration retinal ganglion identifying eleven key genes that accelerate RGC regeneration and seven that inhibited RGC repair. For further study asc1a was chosen because of the surprising finding that KO ascl1a is accelerating RGC repair. Ascl1 has previously been viewed as pro-regenerative in the zebrafish retina and overexpression in mouse MG stimulate repair. To identify further mechanisms a scMultiomics approach was performed on ascl1a KO retina after injury and key regulatory mechanisms and shift in cell bias was identified explaining the accelerated generation of RGCs in ascl1a KO fish. Essentially the ascl1a KO MG becomes "primed" and shift towards the RGC lineage and accelerating fate decision towards RGC production during repair. Overall this is a fantastic study that I have no problems in recommending after minor corrections (see below).

Minor comments

Overall, some attention to text required as text in result is quite succinct.

-In abstract please reword/breakup this sentence in abstract its too complex and confusing

"Here, combining a novel zebrafish model enabling inducible selective cell ablation with single cell multiomics and CRISPR/Cas9-based knockout methods, we screened 100 candidate genes for effects on the regeneration of retinal ganglion cells (RGCs), the cells lost in glaucoma; identifying 7 genes that promote and 11 genes that inhibit RGC regeneration."

-The connection to disease/glaucoma comes a bit abrupt and out of the context in sections/sentences below. Its important to highlight the role of cell types in disease but it really disrupt reading flow as context of text is completely different (regeneration mechanisms). Please move and edit it just doesn't work out well as it is.

In abstract "the regeneration of retinal ganglion cells (RGCs), the cells lost in glaucoma"

Introduction "New RGCs and photoreceptors, the cells most closely associated with retinal disease(11,12), are rarely observed."

-Comment on statement "More recently, overexpression of Ascl1 and Atoh1 resulted in mammalian MG becoming "RGC-like" cells(13), showing cell fate can be modulated to promote the regeneration". Did this study actually show that Ascl1 or Atoh1 programmed "RGC-like" cells become RGC or function like RGC or where they just "RGC-like"?

-In Figure 2. Please increase font size in the pesudotime plots. Its very hard read text among the cells. Please also check that text is readable in other tables/plots

-Relating to CRISPR/Cas9 screen. Four guides were used to target one gene. Were injections targeting only one gene at a time or were several gene targets addressed in one injection? To me appears its one gene/injection is that correct?

© 2024. Published by The Company of Biologists under the terms of the Creative Commons Attribution License (https://creativecommons.org/licenses/by/4.0/).

-Fig S4 A. Is coding sequence referring to transcript/cDNA or genomic/exon?
-Any shared mechanisms between KO genes that improved RGC regeneration? Or the ones inhibiting RGC regeneration? Please comment/dicsuss

-What is causing the accelerated RGC regeneration in the KO fish? Could it be shorter cell cycle, pre-mature/rapid differentiation or priming/biasing MGCPs towards RGC fate? I realise by reading entire manuscript that this is well answered for the ascl1a. Is there any evidence that cells would differentiate it quicker or is it consequence of fate bias?

-The message of this sentence does come through very clearly. Please revise
"Consistent with this, functional tests of the 33 genes known/implicated in regeneration in the context of widespread damage (LD, NMDA, puncture wounds) found that most genes affected only a single retinal regenerative paradigm or had opposing effects in different models, such as ascl1a and sox2 (table S4).

Reviewer 2

Advance summary and potential significance to field

This is a very important manuscript in the field of retinal regeneration that significantly extends our understanding of a very underappreciated aspect of retinal regeneration, namely, how the retina determines which cell type to replace. This has larger implications to retinal development, translational studies in the mouse, and ultimately therapeutic interventions in human retinal degenerative diseases.

Comments for the author

I only have minor editorial comments.

Editorial/typographical
1. "Retinal damage is known to induce Müller glia (MG) to dedifferentiate to a stem-like state and divide to produce a MG-derived progenitor cell (MGPC)."
Lacking nuance and citation. Not all retinal damage triggers MG proliferation in fish. Minimal damage or chronic damage does not (see Perkins/Thummel works)
2. Fig 1e. Unclear why "PCNA+" is italicized here. This appears to be IHC not in situ data
3. "To test if RGCs regenerate following ablation, Mtz-treated RGC:YFP-NTR2 larvae were allowed to recover until 11 dpf (168 hpa, Fig. 1e)." I think this should reference Fig. 1f
4. "Studies using the NTR/Mtz cell ablation system provided initial evidence of "fate biased" retinal regeneration, MGPCs preferentially gave rise to lost cell types following selective amacrine cell and cone photoreceptor ablation(5,6,21)." Not a sentence
5. "A volcano plot of 12 hpa MG DEGs shows two highly induced genes observed in MG single cell data other models of retinal regeneration, c7b(21,26)
and crlf1a(27) (Fig. 2e)." Not a sentence
6. Fig. 2f. It is difficult to assess what timepoint the red asterisks are referring to
7. "However, new cells are generated largely by transdifferentiation of Ascl1-overexpressing MG rather than proliferation and the resulting cell types are limited largely to amacrine and bipolar cells" – clarify that you are referring to the aforementioned mouse study only

Reviewer 3

Advance summary and potential significance to field

Using the highly regenerative zebrafish model, Emmerich et al. demonstrate how cell specific damage directs the retina regenerative response in terms of gene expression and regenerative outcome. This study highlights the importance of the retinal injury-context and the type of response the retina elicits to replace lost neurons. In this paper retinal ganglion cells were genetically ablated and the molecular signatures were monitored using a multiomics approach

© 2024. Published by The Company of Biologists under the terms of the Creative Commons Attribution License (https://creativecommons.org/licenses/by/4.0/).

along with a CRISPRcas screen with surprising results showing key genes previously identified in other injury paradigms (light/chemically induced damage) to play essential roles in the regenerative process, not to be required for RGC regeneration. Instead, genes such as Ascl1, limited the plasticity of muller glia-derived progenitors to differentiate to RGC. This is important work contributing to the field of retina repair and regeneration. Specifically, the RGC Injury paradigm is unique and shows new findings that would help to understand biased regeneration as well as the mechanism involved in cellular fate determination.

Comments for the author

Several revisions are needed for the paper to be on par with the rigor and expectations of this journal.

One critical is that there was no availability to the raw data and pipeline.

The authors suggest that the RGC left after MTZ ablation are most likely displaced amacrine cells. This can be easily addressed by doing an immunohistochemistry against an amacrine marker to ID those cells and more accurately report the efficiency of this line.

For Fig 1 i & j, how do you differentiate endothelial cells in this location with RGC? - it is hard to tell specially in the MTZ treated eyes. Also, what is the red stained region in the dorsal region of the eye in 1i? For readers not familiar with the eye, it would be good to show endothelial cell markers in this line to define that these cells are left behind the RGC layer with MTZ treatment. Also, include a note that lens cells are also proliferative throughout life to explain the + signal- again the readership is broader than just eye researchers.

Discuss the relevance of C7b and Crlfa as these were clearly the most upregulated genes in this paradigm. Any possible functions? KO, no change?
Also address the significance of the upregulation of Sox2, hmga1a, lin28a and stat3 within the 1st 12h in this paradigm vs others- is this a conserved change or unique to RGC ablation?

Can you address the inverse relationship in accessibility between nrl and crx in fig 5 f and g in the ascl1 KO?

Discuss why the transgenic line is limited (challenges encountered and possible insights on how to modify for an adult model) to larvae and juveniles. Using adult fish would be a step closer to what one will encounter during human retinal damage and would be important to address and how a RGC- ablation specific model can be duplicated in an adult model.

Discuss why Atoh7 accessibility increases in Ascl1 KO in the context of this paradigm and how it relates to the work of Le et al. (bioRxiv preprint 2023) highlighting the inhibition of Notch and NF1a factors KO in mice.

The genes that were ID to be essential for RGC regeneration were barely discussed- brushed. This seems to be a critical part of this analysis (from Fig 4c) and important to dissect the mechanisms of RGC regeneration.

The fact that Ascl1a accelerates RGC regeneration is revealing but poorly discussed about its possible mechanism, even when the availability of the data is extensive.

Minor:

1. There is limited reporting on the number of biological samples used in most of the statistical analysis used in this paper and the description of the statistical methodology is limited. For example, in figures 1 b, e, g, j, and l.
Access to raw data is highly recommended. In supplemental fig S1b- it would be best to describe what the comparisons are since there are two bars with significance or ns. Directing the reader on what is being compared would be helpful. **** not defined in fig legend 1 and no description of the significance values on legend for Fig S1.

© 2024. Published by The Company of Biologists under the terms of the Creative Commons Attribution License (https://creativecommons.org/licenses/by/4.0/).

2. Figure 2C: Color coded lines for BC, Rods, HCs could be more obvious or more different than others to follow better
3. Some supplemental figures are not sharp enough to clearly read. Example, Fig S2- X and Y axis descriptions are blurry, especially the gene names.
4. No description of how volcano plot was generated in Fig 2 and n values.
5. Text within UMAPS like #s in subclustering – fig 2 or pseudotime UMAP text – fig 3a and fig 5e is hard to discern- need to be bolded or sharper.
6. Figure legend 3e should say MG>Progenitors> Rods not RGCs.
7. Why are the plots of control RGC and Rod Controls not similar as these retinas were not ablated- Describe if there is a difference or why they are different.
8. Most figures do not describe the pvalues (*) in the legend, except Fig legend # 1.
9. Label Fig S4 "d" with 5 dpf as "c" is labeled with 48 hpa for consistency
10. There are few grammatical errors throughout. For example when referring to multiomic analysis- nuclei were isolated, not cells, therefore should be careful when referring to snRNA/ATACseq and not "sc"; ie page 7: profiling a total of 53,889 cells should be nuclei.
11. Fig 5b- Labels are too small to read, and also it would be nice to have the start site and gene direction to correlate it with the peaks reported (putative promoter/enhancer regions). There is no key color for the values on Fig 5 f and g.
12. Color code- red in Fig 5h is up-regulated but in 5i is down-regulated- it will be good to be consistent on color coding.
13. Ref figures on page 7 at the end of paragraph: "Neurogenic TFs upregulated in ascl1a KO progenitor cells included: crx, foxn4, neurod4, vsx1, otx2b pou2f2a, and atoh7 – and all Sox genes evaluated, except sox2. Strong induction of sox11a/b and sox4a – both implicated in RGC development in mice(38) –
suggests enhanced RGC fate bias in ascl1a KOs. Conversely, neurod1 and nrl showed reduced levels of expression in ascl1 KOs (Fig. 5g), suggesting reduced production of photoreceptors (Fig 5f and g).
14. In the first paragraph of page 9, "this could also account for the pro-RGC regenerative effects of knocking out olig2(49) and possibly neurog". A reference for neurog is missing, and # 49 reference describe the cellular fate of embryonic Olig2+ retinal progenitor cells, and no knockout of olig2 was used.
Other missing references are noted in the manuscript; therefore, it is advisable for the authors to carefully review all references for proper citation and inclusion.
15. define dpa

First revision

Author response to reviewers' comments

We thank the reviewers for their comments and for requesting a more detailed discussion of our results. Their input has inspired us to make a significant number of changes to the original manuscript in response to their specific requests as well as to broaden the discussion overall including an updated title and abstract. To assist with the second review, we will provide two versions for review, one with changes tracked and a second with all changes accepted. Specific responses to reviewer requests are detailed below.

Reviewer 1 Advance summary and potential significance to field
This is an excellently executed study examining how cell type specific ablation in zebrafish retina impacts cell regeneration and compares to broader injury approaches using scRNAseq, multiomics and a substantial amount of CRISPR/Cas9 KO models for validation. The authors selectively ablated retinal ganglion cells (RGCs) using elegant transgenic cell specific reporter and ablation system. Intriguingly, the authors found that Muller Glia (MG), the active progenitor required for repair of the neural retina transcriptionally respond differently to the different injury types. The majority of differentially expressed genes in MG during retinal repair were injury type specific. One hundred candidate genes involved in MG response were selected for CRISPR/Cas9 KO and validated for regeneration retinal ganglion identifying eleven key genes that accelerate RGC regeneration and

© 2024. Published by The Company of Biologists under the terms of the Creative Commons Attribution License (https://creativecommons.org/licenses/by/4.0/).

seven that inhibited RGC repair. For further study asc1a was chosen because of the surprising finding that KO ascl1a is accelerating RGC repair. Ascl1 has previously been viewed as pro-regenerative in the zebrafish retina and overexpression in mouse MG stimulate repair. To identify further mechanisms a scMultiomics approach was performed on ascl1a KO retina after injury and key regulatory mechanisms and shift in cell bias was identified explaining the accelerated generation of RGCs in ascl1a KO fish. Essentially the ascl1a KO MG becomes "primed" and shift towards the RGC lineage and accelerating fate decision towards RGC production during repair. Overall this is a fantastic study that I have no problems in recommending after minor corrections (see
below).

Minor comments

Overall, some attention to text required as text in result is quite succinct.

-In abstract please reword/breakup this sentence in abstract its too complex and confusing

"Here, combining a novel zebrafish model enabling inducible selective cell ablation with single cell multiomics and CRISPR/Cas9-based knockout methods, we screened 100 candidate genes for effects on the regeneration of retinal ganglion cells (RGCs), the cells lost in glaucoma; identifying 7 genes that promote and 11 genes that inhibit RGC regeneration."

Response: We have updated this and other elements of the abstract to improve clarity and stay within word limits. See updated version below (a version with all changes tracked is also uploaded).

Many genes are known to regulate retinal regeneration following widespread tissue damage. Conversely, genes controlling regeneration following limited cell loss, per degenerative diseases, are undefined. As stem/progenitor cell responses scale to injury levels, understanding how the extent and specificity of cell loss impact regenerative processes is important. Here, transgenic zebrafish enabling selective retinal ganglion cell (RGC) ablation were used to identify genes that regulate RGC regeneration. A single cell multiomics-informed screen of 101 genes identified seven knockouts that inhibited and eleven that promoted RGC regeneration. Surprisingly, 35 of 36 genes known/implicated as being required for regeneration following widespread retinal damage were not required for RGC regeneration, and seven even enhanced regeneration kinetics, including proneural factors neurog1, olig2, and ascl1a. Mechanistic analyses revealed ascl1a disruption increased the propensity of progenitor cells to produce RGCs; i.e., increased "fate bias". These data demonstrate plasticity in how Müller glia can convert to a stem-like state and context-specificity in how genes function during regeneration. Increased understanding of how the regeneration of disease-relevant cell types is specifically controlled will support the development of disease-tailored regenerative therapeutics.

-The connection to disease/glaucoma comes a bit abrupt and out of the context in sections/sentences below. Its important to highlight the role of cell types in disease but it really disrupt reading flow as context of text is completely different (regeneration mechanisms). Please move and edit it just doesn't work out well as it is.

In abstract "the regeneration of retinal ganglion cells (RGCs), the cells lost in glaucoma"

Response: deleted (see above)

Introduction "New RGCs and photoreceptors, the cells most closely associated with retinal disease(11,12), are rarely observed."

Response: Updated, see changes below.

We were particularly intrigued by the ascl1a result as forced expression of Ascl1 in mouse MG cells leads to the production of new neurons following retinal damage (Jorstad et al., 2017; Ueki et al., 2015). Unfortunately, Ascl1 expression alone does not promote substantial production of the retinal cell types most relevant to disease, RGCs and photoreceptors.

© 2024. Published by The Company of Biologists under the terms of the Creative Commons Attribution License (https://creativecommons.org/licenses/by/4.0/).

-Comment on statement “More recently, overexpression of Ascl1 and Atoh1 resulted in mammalian MG becoming “RGC-like” cells(13), showing cell fate can be modulated to promote the regeneration”. Did this study actually show that Ascl1 or Atoh1 programmed “RGC-like” cells become RGC or function like RGC or where they just “RGC-like”?

Response: We are deferring to the authors of those studies on this point by using their terminology, “RGC-like”. The authors use this term to indicate that these cells do not fully differentiate into mature RGCs. They speculate that this may be due to persistent expression of Ascl1 which holds the cells in a “mixed” state, expressing markers of both progenitor cells and immature RGCs. This possibility is now detailed in an expanded Intro section, where we also discuss how these cells were determined to be “RGC-like”.

The revised Introduction section now reads:

However, more recently, overexpression of Ascl1 and Atoh1 was shown to be sufficient for mouse MG producing “RGC-like” cells, i.e., exhibiting some features of RGCs, such as production of action potentials, but sharing a transcriptomic signature with retinal progenitors and early amacrine cells, indicating a lack of full RGC maturation (Pavlou et al., 2024; Todd et al., 2021; Todd et al., 2022) . These data show that cell fate can be modulated to promote the regeneration of disease-relevant cell types in the mammalian retina, and highlight the need to better understand how proliferation and cell fate are controlled during retinal regeneration.

-In Figure 2. Please increase font size in the pesudotime plots. Its very hard read text among the cells. Please also check that text is readable in other tables/plots

Response: Done.

-Relating to CRISPR/Cas9 screen. Four guides were used to target one gene. Were injections targeting only one gene at a time or were several gene targets addressed in one injection? To me appears its one gene/injection is that correct?

Response: For the majority of cases a single gene was targeted. In cases where paralogs have arisen due to genome duplication, a total of eight gRNAs were injected, four targeting each paralog. We have now added this detail to the Results, the Materials and Methods and in a Table of the gRNAs used. See also Tables S4 and S5

Results: “All genes were targeted individually or as pairs (for paralogs).”

Materials and Methods: “When paralogs of the targeted gene were present, both paralogs were targeted by injecting four sgRNAs per paralog; e.g., a total of eight sgRNAs when targeting two paralogs.”

-Fig S4 A. Is coding sequence referring to transcript/cDNA or genomic/exon?
-Any shared mechanisms between KO genes that improved RGC regeneration? Or the ones inhibiting RGC regeneration? Please comment/dicsuss

Responses:
1) For Fig. S4A, coding sequence refers to genomic DNA. We have updated the figure legend so that this is clear.

2/3) Shared mechanisms? We have no concrete mechanistic data to support this, but it is an active area of follow on research in the lab. In the absence of conclusive data, we limit discussion of this issue to possible explanations derived from prior studies of gene function for the factors we identified. New text was added to the Discussion for points a-d to provide further context into potential shared mechanisms:
a. Prevalence of transcription factors among gene KOs the improved RGC regeneration, four bHLH and one SYR-box. Previously, we had discussed the possibility of dose-dependent effects of sox2 on RGC differentiation. We now include additional details regarding potential mechanisms of olig2 and neurog1 The following updates were added to address point (a):

© 2024. Published by The Company of Biologists under the terms of the Creative Commons Attribution License (https://creativecommons.org/licenses/by/4.0/).

Among 11 crispants that accelerated RGC regeneration kinetics, i.e., anti-RGC regeneration factors, five of the targeted genes are transcription factors, three basic helix-loop-helix (bHLH) proneural factors (ascl1a, olig2, neurog1), one SRY-box factor that regulates the expression of proneural genes such as neurog2 and neurod1 (sox2; (Amador-Arjona et al., 2015)) and one bHLH predicted to regulate the cell cycle (max; (Jones, 2004)).

…Increased fate bias could also account for the pro-RGC regenerative effects of knocking out two other bHLH transcription factors, olig2 and neurog1. Olig2-expressing mouse retinal progenitors, like Ascl1-expressing progenitors, rarely produce RGCs (Hafler et al., 2012). Similarly, loss of Neurog1 increases early-born fates in the mouse cortex (Han et al., 2018), suggesting neurog1 crispants may similarly increase RGCs as they are one of the earliest born neurons in the retina. The effect of sox2 disruption may be dosage dependent, with low levels of expression promoting precocious RGC differentiation via upregulation of atoh7 (formerly ath5) and inhibition of Notch (Taranova et al., 2006).
b. Prevalence of mediators of the unfolded protein response (UPR), hspd1, hsp90b1, and atf6, among genes predicted to be pro-RGC regeneration factors.
c. Circadian genes as potential positive regulators of RGC regeneration: bmal2, cry3b (formerly arntl2b and cry2b).
d. Possibility that KO of mlc1, a target of Notch/Rbpj signaling, promoted MG dedifferentiation to promote retinal regeneration.
The following updates were added to the Discussion for points (b-d):
Combining a novel transgenic line enabling targeted RGC ablation with single cell multiomics and large-scale reverse genetic screening, we identified 18 genes whose disruption altered RGC regeneration kinetics. Of the 7 crispants that inhibited RGC replacement most have no known role in regeneration, except hspd1 (Qin et al., 2009) (above) and hsp90b1 (i.e., effects on senescence during muscle regeneration (He et al., 2019)). These genes are intriguing as their normal function is predicted to promote RGC regeneration. KO of mlc1, a target of Notch/Rbpj signaling, may promote MG dedifferentiation to promote regeneration. Three other pro-RGC regeneration genes are involved in mediating the unfolded protein response (UPR), hspd1, atf6, and hsp90b1, and two involved in mediating circadian rhythmicity, bmal1 and cry3b. These results suggest reduction of UPR-mediated cell stress may promote regeneration in general as this pathway has also been implicated in hair cell and tail regeneration (Lin et al., 2021; Pei et al., 2018) and add to prior evidence of circadian gene involvement in retinal regeneration (Qin et al., 2009).

-What is causing the accelerated RGC regeneration in the KO fish? Could it be shorter cell cycle, pre-mature/rapid differentiation or priming/biasing MGCPs towards RGC fate? I realise by reading entire manuscript that this is well answered for the ascl1a. Is there any evidence that cells would differentiate it quicker or is it consequence of fate bias?

Responses: These are key questions and, as mentioned above, we are actively investigating “how” RGC regeneration is accelerated for the remaining 17 genes implicated. For ascl1a, the data are clear, no difference in proliferation was observed but a clear reduction in non-RGC cell production was evident. As discussed throughout the manuscript, we interpreted this result as enhanced fate bias, i.e., an increase in the propensity of MGPCs to give rise to RGCs. This interpretation is consistent with the role of ascl1 during development in the mouse retina: Ascl1-expressing progenitors give rise predominantly to all retinal cell types except RGCs. By removing this differentiation path, the likelihood of progenitors producing RGC was increased. To expand on this theme of the manuscript, we have added a discussion of recent and prior publications that have reached contrasting conclusions regarding the degree to which the nature of retinal injury/cell loss influences MGPC fates – thus the capacity of MGPCs to exhibit fate bias. The following changes and added text are included in the revised Discussion:
Our results demonstrate plasticity in how MG convert to a stem cell-like state and context specificity in how genes functions to regulate retinal regeneration, that is, ascl1a-independent mechanisms of MG dedifferentiation and a preponderance of paradigm-specific gene effects, respectively. These findings echo emerging evidence of divergence in how retinal regeneration is regulated across paradigms, suggesting regenerative processes are informed by and adapt to the nature of the injury incurred (Emmerich et al., 2023b; Lyu et al., 2023). Where this information originates (dying and/or surviving cells), how it is transmitted, and how it controls MGPC proliferation rates and cell fate decisions are largely unresolved.

© 2024. Published by The Company of Biologists under the terms of the Creative Commons Attribution License (https://creativecommons.org/licenses/by/4.0/).

… In a recent comparison of LD and NMDA excitotoxicity in zebrafish, Lyu et al., made several key observations that further support the hypothesis that the nature of the injury informs retinal regenerative processes and which underscore key differences between retinal development and retinal regeneration (Lyu et al., 2023)These insights explain why MGPC responses to LD and NMDA injury paradigms have been previously characterized as “multipotent” (Powell et al., 2016) rather than responsive to injury specifics: a higher degree of cell death specificity had been assumed. Moreover, the data from Lyu et al., further support the idea that MGPCs can exhibit fate bias, clarifying that even when cell death is widespread throughout all layers of the retina, MGPCs are exquisitely attuned to the nature of the injury. In further support of this concept, single cell transcriptomics analyses revealed divergent gene expression across retinal regeneration paradigms; ~70% of MG DEGs were paradigm-specific rather than shared across RGC ablation and LD/NMDA models. Similarly, a comparison of two selective retinal cell ablation models targeting either rod photoreceptor or retinal bipolar cells, showed paradigm-specific gene changes predominated in bulk RNA datasets (Emmerich et al., 2023b).

In contrast to these findings, a recent report has shown that the type of injury incurred, either LD or NMDA, does not alter cell fate in the context of Ascl1 overexpressing mouse MG cells (Pavlou et al., 2024). Interestingly, when Atoh1 and Ascl1 are co-expressed in mouse MG, the type of injury incurred does have a small effect on the types of cells generated – 80% RGC-like versus 90% RGC-like in LD versus NMDA, respectively. Given the findings of Lyu et al., that neither LD nor NMDA-based damage paradigms induce layer-specific cell losses in zebrafish, it will be prudent to re-evaluate the specificity of cell death across all current paradigms by evaluating cell loss acutely and in subsequent days to account for secondary “bystander” cell death. A simple explanation for the seeming discrepancy between the findings of Pavlou et al. and “fate biased” regenerative responses observed in zebrafish is that they are due to technical differences. That is, the effects of forced expression of proneural transcription factors in mouse MG may not reflect the natural course of regeneration in zebrafish. Our interpretation of the ascl1a KO zebrafish data is, in fact, not inconsistent with forced Ascl1a and/or Ascl1a-Atoh1 expression having relatively invariant effects on the fate of MGPCs in mice – i.e., we would predict that this would bias MGPC toward fates specified by the overexpressed factor(s).

To test for context-specific gene function during retinal regeneration more comprehensively, 36 previously identified/implicated regulators of retinal regeneration following widespread tissue damage were screened for effects on regeneration following selective RGC ablation. The majority of tested factors, 16 genes previously shown to be required for and 20 genes implicated in retinal tissue regeneration, had either no statistically significant effect (28 genes) or accelerated RGC regeneration kinetics (7 genes). Only one crispant showed concordant effects across paradigms, hspd1 KO inhibiting regeneration after LD (Qin et al., 2009) and RGC ablation. In addition, a gene we previously implicated in retinal bipolar cell regeneration, pparg, had no effect on either rod photoreceptor (Emmerich et al., 2023b) or RGC regeneration. We also saw no effects on RGC regeneration upon targeting JAK/STAT signaling (stat3, stat5a/b, jak1), which differs from results in retinal damage (Elsaeidi et al., 2014; Nelson et al., 2012; Todd et al., 2016; Zhang et al., 2005) and selective rod cell ablation models (Emmerich et al., 2023b). These results highlight the need for functional gene testing across different regenerative models to account for paradigm-specific effects.

-The message of this sentence does come through very clearly. Please revise “Consistent with this, functional tests of the 33 genes known/implicated in regeneration in the context of widespread damage (LD, NMDA, puncture wounds) found that most genes affected only a single retinal regenerative paradigm or had opposing effects in different models, such as ascl1a and sox2 (table S4).

Response:
Response:
Agreed. In light of: 1) this comment, 2) requests from reviewers 2 and 3 for further elaboration/discussion of crispant screen results (see below), and 3) our initial submission being under the 7,000 word limit for Research Articles, we have elected to substantially expand on details provided in Result and Discussion. Our intent is to provide a more comprehensive overview of the data and the implications of our interpretation of the finding. In addition to substantial

© 2024. Published by The Company of Biologists under the terms of the Creative Commons Attribution License (https://creativecommons.org/licenses/by/4.0/).

amount of added data details, associated Figures/Tables were updated to clarify the outcomes of the screen and of functional comparisons across retinal regeneration paradigms. In addition, we now discuss 14 additional genes that trended toward significant effects (FDR adj. p-value of >0.01 to 0.1), bringing the total number of genes implicated in RGC regeneration to 32. Finally, to avoid confusion about the numbers of genes tested, we sum all paralogs tested as 2 genes rather than one (thus the difference in final numbers from the initial manuscript). Changes within the expanded Results section are provided below:

Reverse genetics “crispant” screen: knockout of ascl1a enhances RGC regeneration
To identify regulators of RGC regeneration, we performed a large-scale reverse genetics screen. An efficient CRISPR/Cas9-based method (Wu et al., 2018) was used to create biallelic mutations in 101 genes of interest. All genes were targeted individually or as pairs (for paralogs). Briefly, fertilized RGC:YFP-NTR2 eggs were co-injected with Cas9 and four gRNAs per targeted gene. This approach induces widespread somatic mutation of the targeted loci, enabling phenotypic screens with the injected “crispant” fish (Shaw and Mokalled, 2021). Crispant larvae and controls were treated with 100 μM Mtz from 5-6 dpf. Plate reader reader-based quantification of YFP was then used to screen for effects on RGC regeneration kinetics at 9 dpf (4 dpa), a time point where ~32% of RGCs had regenerated in control wildtype larvae (Fig. 4a,b; note that plate reader assays quantify both cellular and axonal YFP content, thus the difference in RGC kinetics relative to confocal imaging of RGC axons only, Fig. 1F,G). 62 candidate genes were chosen from our scRNA-seq dataset. Another 39 genes were selected from the literature (Hoang et al., 2020) based on being either previously shown to be required for retinal regeneration following widespread retinal damage (19 genes) or strongly implicated as being required in that process (20 genes; Tables S4 and S5). Ten crispant KOs produced developmental defects that precluded testing effects on RGC regeneration (3 from the known/implicated set – hmga1a, yap1, tgif1 – and 7 from the scRNA-seq set – bax, fax2a, ctgfa, mcf2a, smarca5, xbp1, bmp2b; Table S4). Most viable crispants were similar to controls, with 73 of 91 candidates having no significant effect on RGC regeneration kinetics (Fig. 4c, Table S4; statistical significance was defined as a FDR adjusted p-value of >0.01). Among these, 28 came from the 39 genes previously known/implicated in retinal tissue regeneration and another 45 came from the 62 genes selected from our scRNA-seq dataset (Fig. 4c, Table S4).

Of the remaining 18 genes, eleven crispants exhibited enhanced RGC replacement kinetics (ascl1a, neurog1, max, sox2, olig2, lepb, tubb4b, nfasca, tnfrsf11b, fosab, and mink1) while KO of seven others inhibited RGC regeneration (slc1a2b, hspd1, cry3b, mlc1, bmal2, hsp90b1, and atf6; Fig. 4c, Table S4). However, five additional KOs trended toward increased regenerative kinetics (ptmab, rbp4l, tyr, lin28ab, and klf14), and nine others trended toward inhibition of RGC regeneration (ctnnb1, zgc:112334, met, cahz, efhd1, isl1a, aqp1a.1, mcf2a, jak1; Fig. 4c, Table S4; trending defined as an FDR adjusted p-value of >0.01 to 0.1). Including the trending factors, 32 genes were implicated as regulators of RGC regeneration: 16 pro-regenerative factors (7 significant and 9 trending) and 16 anti-regenerative factors (11 significant and 5 trending; note that predicted gene functions are the opposite of KO effects, thus the reversal of effect here). Of these, 11 were previously implicated in retinal regeneration and 21 are novel (Table S4).

Interestingly, in comparing the effects of known/implicated regulators on retinal tissue regeneration we noted that nearly all of them exhibited discordant effects on RGC regeneration: either having no effect (28 genes) or opposite effects (7 genes) (Tables S4 and S5). For example, KO of mmp9 had no effect on RGC regeneration, despite having roles in promoting INL and GCL fates in LD and NMDA paradigms, respectively (Table S5; (Lyu et al., 2023)). Similarly, sox2 KOs showed pro-regenerative effects in the RGC paradigm but produced anti-regenerative effects in the context of LD, and overexpression led to increased proliferation in uninjured control retinas (Gorsuch et al., 2017). Additional genes where disruption led to accelerated RGC regeneration kinetics included ascl1a (Fausett et al., 2008), olig2 (Fimbel et al., 2007), and lepb (Zhao et al., 2014). Even among 19 genes previously shown to be “required” for retinal regeneration in the context of tissue damage paradigms, 13 had no effect, two KOs promoted (KO of lin28 also trended toward a pro-regenerative effect), and one KO inhibited RGC regeneration (three caused developmental defects that precluded testing; Fig. 4c, Tables S4 and S5). The only gene exhibiting a concordant effect across paradigms was hspd1, where KO caused anti-regenerative effects in RGC and LD paradigms (Qin et al., 2009). In all, 35 of 36 known/implicated regulators of retinal regeneration following widespread tissue damage (LD, NMDA, puncture wound) had either no or opposing effects on RGC regeneration (Tables S4 and S5).

© 2024. Published by The Company of Biologists under the terms of the Creative Commons Attribution License (https://creativecommons.org/licenses/by/4.0/).

We were particularly intrigued that KO of ascl1a led to accelerated RGC regeneration. This gene was initially selected as our control for inhibiting RGC replacement due to the widespread notion that it is “required” for retinal regeneration (Fausett et al., 2008). Moreover, forced expression of Ascl1 in mouse MG stimulates a nascent regenerative response in the injured mouse retina (Todd et al., 2021; Todd et al., 2022). Follow up plate reader assays (Fig. 4d) as well as in vivo imaging (Fig. 4e) confirmed primary screen results, showing that KO of ascl1a enhanced RGC regeneration. Additional controls showed CRISPR/Cas9-based KO of ascl1a was highly efficient, had no effect on RGC development, or Mtz-induced RGC death (Fig. S4). To assess specificity, we tested the effect of ascl1a KO on rod photoreceptor regeneration kinetics and saw no change by either plate reader assay (Fig. 4f) or intravital imaging (Fig. 4g).

Reviewer 2 Advance summary and potential significance to field
This is a very important manuscript in the field of retinal regeneration that significantly extends our understanding of a very underappreciated aspect of retinal regeneration, namely, how the retina determines which cell type to replace. This has larger implications to retinal development, translational studies in the mouse, and ultimately therapeutic interventions in human retinal degenerative diseases.

Reviewer 2 Comments for the author
I only have minor editorial comments.

Editorial/typographical
1.“Retinal damage is known to induce Müller glia (MG) to dedifferentiate
to a stem-like state and divide to produce a MG-derived progenitor cell (MGPC).” Lacking nuance and citation. Not all retinal damage triggers MG proliferation in fish. Minimal damage or chronic damage does not (see Perkins/Thummel works)

Response:
Thank you for noting this lack of nuance. We agree it is important to add an appropriate level of detail here as our results, and data from related studies, suggests the regenerative response scales to the level and specificity of the injury incurred, including control over progenitor cell fate decisions. To ensure clarity and provide appropriate citations this section, and a similar section of the Introduction which also lacked nuance and appropriate references, were updated. The changes made are shown below.

Introduction :
Insights into the genes that regulate retinal regeneration in zebrafish have come almost exclusively from acute widespread retinal injury paradigms such as puncture wounds (Sharma and Ramachandran, 2022), light damage (LD (Lahne et al., 2020a; Lenkowski and Raymond, 2014), and chemical toxins (e.g., NMDA; (Hoang et al., 2020)). A recent report has clarified that both LD and NMDA are not specific to the targeted cell layer but, like puncture wounds, result in substantial cell death throughout all retinal cell layers (Lyu et al., 2023). Thus, current understanding of the factors controlling retinal regeneration must be framed in the context of replacing the retina as a tissue. In contrast, factors controlling regeneration following selective retinal cell loss, a hallmark of retinal degenerative disease, are largely unknown. We posited that accounting for how disease-relevant parameters, such as the extent and specificity of retinal cell death, impact the regenerative process will be important for the development of disease-tailored regenerative therapeutics. Accordingly, we designed a study to identify genes that regulate the regeneration of retinal ganglion cells (RGCs), the cells lost in glaucoma, following selective RGC loss.

Results:
Extensive acute retinal damage is known to induce Müller glia (MG) to dedifferentiate to a stem-like state (Bernardos et al., 2007; Fausett et al., 2008; Fimbel et al., 2007) and divide asymmetrically to produce a MG-derived progenitor cell (MGPC; (Bernardos et al., 2007)). MGPCs are transit amplifying neural progenitor cells which divide further and differentiate into new retinal neurons (Goldman, 2014; Lahne et al., 2020b; Nagashima et al., 2013).

2.Fig 1e. Unclear why “PCNA+” is italicized here. This appears to be IHC, not in situ data

© 2024. Published by The Company of Biologists under the terms of the Creative Commons Attribution License (https://creativecommons.org/licenses/by/4.0/).

Response: Typo, we fixed the PCNA label in a revised Fig. 1

3.“To test if RGCs regenerate following ablation, Mtz-treated RGC:YFP-NTR2 larvae were allowed to recover until 11 dpf (168 hpa, Fig. 1e).” I think this should reference Fig. 1f

Response: revised text reads Fig. 1f

4.“Studies using the NTR/Mtz cell ablation system provided initial
evidence of “fate biased” retinal regeneration, MGPCs preferentially gave rise to lost cell types following selective amacrine cell and cone photoreceptor ablation(5,6,21).” Not a sentence

Response: revised to, “Studies using the NTR/Mtz cell ablation system provided initial evidence of “fate biased” retinal regeneration, with MGPCs preferentially giving rise to the lost cell types following selective amacrine cell or cone photoreceptor ablation (D’Orazi et al., 2020; Emmerich et al., 2023b; Ng Chi Kei et al., 2017).”

5.“A volcano plot of 12 hpa MG DEGs shows two highly induced genes
observed in MG single cell data other models of retinal regeneration, c7b(21,26) and crlf1a(27) (Fig. 2e).” Not a sentence

Response: Thanks, revised to, “A volcano plot of 12 hpa MG DEGs shows two immune-related factors, c7b (Emmerich et al., 2023b; Sifuentes et al., 2016) and crlfa (Laura et al., 2023), are the most highly upregulated genes in MG upon RGC ablation (Fig. 2e).“

6.Fig. 2f. It is difficult to assess what timepoint the red asterisks are referring to

Response: Updated Fig. 2f to make time points of interest clearer

7.“However, new cells are generated largely by transdifferentiation of
Ascl1-overexpressing MG rather than proliferation and the resulting cell types are limited largely to amacrine and bipolar cells” – clarify that you are referring to the aforementioned mouse study only

Response: Personal communication from these authors has clarified that this limitation was technical in nature – revised labeling protocols now show robust MG/MGPC proliferative responses. In light of this we have eliminated all references to the “transdifferentiation” limitation of mouse studies. The text has been revised to:

We were particularly intrigued by the ascl1a result as forced expression of Ascl1 in mouse MG cells leads to the production of new neurons following retinal damage (Jorstad et al., 2017; Ueki et al., 2015). Unfortunately, Ascl1 expression alone does not promote substantial production of the retinal cell types most relevant to disease, RGCs and photoreceptors. However, more recently, overexpression of Ascl1 and Atoh1 was shown to be sufficient for mouse MG producing “RGC-like” cells, i.e., exhibiting some features of RGCs, such as production of action potentials, but sharing a transcriptomic signature with retinal progenitors and early amacrine cells, indicating a lack of full RGC maturation (Pavlou et al., 2024; Todd et al., 2021; Todd et al., 2022) . These data show that cell fate can be modulated to promote the regeneration of disease-relevant cell types in the mammalian retina, and highlight the need to better understand how proliferation and cell fate are controlled during retinal regeneration.

***** Reviewer 3 Advance summary and potential significance to field
Using the highly regenerative zebrafish model, Emmerich et al. demonstrate how cell specific damage directs the retina regenerative response in terms of gene expression and regenerative outcome. This study highlights the importance of the retinal injury-context and the type of response the retina elicits to replace lost neurons. In this paper retinal ganglion cells were genetically ablated and the molecular signatures were monitored using a multiomics approach along with a CRISPRcas screen with surprising results showing key genes previously identified in other injury paradigms (light/chemically induced damage) to play essential roles in the regenerative process, not to be required for RGC regeneration. Instead, genes such as Ascl1, limited the plasticity of muller glia-derived progenitors to differentiate to RGC. This is important work contributing to the field of retina repair and regeneration. Specifically, the RGC Injury

© 2024. Published by The Company of Biologists under the terms of the Creative Commons Attribution License (https://creativecommons.org/licenses/by/4.0/).

paradigm is unique and shows new findings that would help to understand biased regeneration as well as the mechanism involved in cellular fate determination.

Reviewer 3 Comments for the author

Several revisions are needed for the paper to be on par with the rigor and expectations of this journal.

One critical is that there was no availability to the raw data and pipeline.

Response: Raw and processed sequencing data supporting the findings in this study are available online at Gene Expression Omnibus at accession GSE268179: https://www.ncbi.nlm.nih.gov/geo/query/acc.cgi?acc=GSE268179.

The authors suggest that the RGC left after MTZ ablation are most likely displaced amacrine cells. This can be easily addressed by doing an immunohistochemistry against an amacrine marker to ID those cells and more accurately report the efficiency of this line.

Response: The presence of a substantial number of displaced amacrine cells (DACs) in the ganglion cell layer of the larval zebrafish retina is well documented, including prior work we were involved with (Kay et al., Development, 2004) . Given that fact, we ask that we be allowed to add a reference where DAC and RGC numbers were quantified in a 7 dpf larval zebrafish retina (https://www.ncbi.nlm.nih.gov/pmc/articles/PMC7416113/; Zhou et al., Curr Biol 2020) to support our interpretation of the data.

For Fig 1 i & j, how do you differentiate endothelial cells in this location with RGC? - it is hard to tell specially in the MTZ treated eyes. Also, what is the red stained region in the dorsal region of the eye in 1i? For readers not familiar with the eye, it would be good to show endothelial cell markers in this line to define that these cells are left behind the RGC layer with MTZ treatment. Also, include a note that lens cells are also proliferative throughout life to explain the + signal- again the readership is broader than just eye researchers.

Response: EdU+ vascular endothelial cells were delineated by their presence immediately interior to the ganglion cell layer and their distinctive elongated nuclei, a well-established morphological hallmark of these cells (https://www.ncbi.nlm.nih.gov/pmc/articles/PMC4841761/; Hedberg-Buenz et al., Exp Eye Res, 2016 and (Cuthbertson and Mandel 1986, Schallek, Geng et al. 2013)). These details and references were added to the Materials and Methods section to clarify the criteria used for cell type classifications. EdU staining at the margins of the retina delineate the ciliary margin zone, a perennially proliferative region responsible for annular growth of the retina. The “red stained region in the dorsal region of the eye in 1i” – which we assume refers to the +Mtz image on the right – is EdU staining within the brain, not the retina, which is routinely observed when assessing proliferation at this age. We have added this information to the figure legend.

Discuss the relevance of C7b and Crlfa as these were clearly the most upregulated genes in this paradigm. Any possible functions? KO, no change? Also address the significance of the upregulation of Sox2, hmga1a, lin28a and stat3 within the 1st 12h in this paradigm vs others- is this a conserved change or unique to RGC ablation?

Response: 1) We expanded the discussion of c7b and crlfa by making the following changes to the Results, “A volcano plot of 12 hpa MG DEGs shows two immune-related factors, c7b (Emmerich et al., 2023b; Sifuentes et al., 2016) and crlfa (Laura et al., 2023), are the most highly upregulated genes in MG upon RGC ablation (Fig. 2e).”
We also added the following to the Discussion to clarify results of functional tests:
“Our lab and others have recently shown that the immune system plays a critical role in regulating retinal regeneration (Emmerich et al., 2023a; Lyu et al., 2023; Nagashima and Hitchcock, 2021; White et al., 2017). This is consistent with immune-related factors being among the most highly upregulated genes in MG during initial phases of the response to retinal injury (Laura et al., 2023; Sifuentes et al., 2016). In addition, several immune signaling associated factors were among the genes whose disruption altered RGC regeneration kinetics, including hspd1, hsp90bl, atf6 (among

© 2024. Published by The Company of Biologists under the terms of the Creative Commons Attribution License (https://creativecommons.org/licenses/by/4.0/).

inhibitory crispants) and lepb, tnfsrf11b (among accelerated crispants). Similarly, we recently found that c7b crispants exhibit accelerated rod photoreceptor regeneration kinetics (Emmerich et al., 2023b). Studies in which IL6 family member expression was modulated, including the alternative CNTF receptor ligand gene crlf1a, demonstrate that immune signaling also controls optic nerve regeneration in zebrafish; RGC axon regrowth following optic nerve crush is inhibited in crlf1a morphants (Elsaeidi et al., 2014). However, here, KO of c7b or crlf1a had no effect on RGC regeneration (Table S4). These results suggest immune-mediated regulation of retinal regeneration is also context-specific, with immune-related genes having potentially discordant role(s) across regeneration paradigms."
2) We provide further context for the early expression changes observed for sox2, hmga1a, lin28a and stat3, in the Discussion: "Other MG activation markers enriched in 12 hpa MG included sox2, hmga1a, lin28ab, and stat3 (Fig. 2f). These changes are consistent with bulk RNA-seq data from purified MG following acute LD (Sifuentes et al., 2016) and/or single cell data from LD and NMDA paradigms (Hoang et al., 2020).

Can you address the inverse relationship in accessibility between nrl and crx in fig 5 f and g in the ascl1 KO?

Response: We have added nuance to the discussion of this result. Previously we said, "Conversely, neurod1 and nrl showed reduced levels of expression in ascl1 KOs (Fig. 5g), suggesting reduced production of photoreceptors". This is consistent with MGPCs shifting toward early neuronal fates in the absence of ascl1a. We now hypothesize that upregulation of crx in ascl1a KO is consistent with MGPCs exiting from the cell cycle early and/or increased MGPC differentiation into cone photoreceptors (due to the downregulation of nrl), both of which are consistent with MGPCs shifting toward early neuronal fates in the absence of ascl1a. The updated text reads, "Conversely, neurod1 and nrl showed reduced levels of expression in ascl1 Kos (Fig. 5f), suggesting reduced production of photoreceptors (Mears et al., 2001; Ochocinska and Hitchcock, 2009). The increased expression of crx in ascl1a KO MGPCs suggests they exit the cell cycle early (Muranishi et al., 2011) and/or increased cone photoreceptor differentiation (due to the downregulation of nrl). Both of these possibilities are consistent with MGPCs shifting toward early neuronal fates in the absence of ascl1a."

Discuss why the transgenic line is limited (challenges encountered and possible insights on how to modify for an adult model) to larvae and juveniles. Using adult fish would be a step closer to what one will encounter during human retinal damage and would be important to address and how a RGC- ablation specific model can be duplicated in an adult model.

Response: We have added a discussion of strategies that could be used to overcome downregulation of the isl2b:Gal4 driver line and/or silencing of the UAS:YFP-2A-NTR2 reporter/effector line – both of which are potential explanations for the loss of expression in adults that limit current resources.

Discuss why Atoh7 accessibility increases in Ascl1 KO in the context of this paradigm and how it relates to the work of Le et al. (bioRxiv preprint 2023) highlighting the inhibition of Notch and NF1a factors KO in mice.

Response: 1) We have added a discussion of ATAC-seq data to underscore our interpretation that ascl1a KO leads to an increased "RGC fate bias" in MGPCs. Increased Atoh7 accessibility is consistent with that. 2) We do not feel a comparison to the Le et al., study is warranted as the two paradigms under study are fundamentally different – induction of transdifferentiation of mouse MG into neurons versus induction of zebrafish MG to stem cell-like state, asymmetric cell division to produce MGPCs, MGPC expansion as a transit amplifying population and MGPC differentiation into neurons. Moreover, Le et al., did not discuss changes in expression or accessibility of atoh7 in their manuscript. For these reasons, we feel any comparison of how these processes are regulated would be clouded by proverbial apples to oranges caveats.

The genes that were ID to be essential for RGC regeneration were barely discussed- brushed. This seems to be a critical part of this analysis (from Fig 4c) and important to dissect the mechanisms of RGC regeneration.

© 2024. Published by The Company of Biologists under the terms of the Creative Commons Attribution License (https://creativecommons.org/licenses/by/4.0/).

Response: We have expanded the discussion of the factors predicted to mediate pro-regenerative effects (i.e., crispants where RGC regeneration was inhibited). The updated text, with changes tracked, reads: “We identified 18 genes whose disruption altered RGC regeneration kinetics. Of the 7 crispants that inhibited RGC replacement most have no known role in regeneration, except hspd1 (39) (above) and hsp90b1 (i.e., effects on senescence during muscle regeneration (44)). These genes are intriguing as their normal function is predicted to promote RGC regeneration. Among these pro-RGC regeneration factors were three genes involved in mediating the unfolded protein response (UPR), hspd1, atf6, and hsp90b1, and two involved in mediating circadian rhythmicity, bmal1 and cry3b. These results suggest reduction of UPR-mediated cell stress may promote regeneration in general as this pathway has also been implicated in hair cell and tail regeneration (Lin et al., 2021; Pei et al., 2018) and add to prior evidence of circadian gene involvement in retinal regeneration (Qin et al., 2009).”

The fact that Ascl1a accelerates RGC regeneration is revealing but poorly discussed about its possible mechanism, even when the availability of the data is extensive.

Response: To further detail potential mechanisms by which loss of ascl1a biased MGCs toward RGC fates, we have expanded the discussion of proneural TF gene expression hcnages (see Discussion of olig2 and neurog1, above) and our scATAC-seq and pseudotime results from ascl1a KOs. The following has been added to the Discussion:

The ascl1a KO scATAC-seq data support this explanation, showing increased accessibility for: 1) TFs associated with RGC production and/or early retinal cell fates, atoh7 (Kay et al., 2001), neurod2 (Cherry et al., 2011), and sox4 (Chang et al., 2017; Jiang et al., 2013); 2) TFs whose overexpression promotes RGC-like production in mice, atoh1 (Todd et al., 2021; Todd et al., 2022), and 3) known retinal stem/progenitor cell markers, otx1 (Diacou et al., 2022) six3 (Raven et al., 2018), e2f2 (Dagnino et al., 1997) and nr2f2 (Tang et al., 2010). Similarly, pseudotime data shows upregulation of additional TFs that drive RGC fate, including sox11a/b (Chang and Hertz, 2017; Chang et al., 2017). Increased fate bias could also account for the pro-RGC regenerative effects of knocking out two other bHLH transcription factors, olig2 and neurog1. Olig2-expressing mouse retinal progenitors, like Ascl1-expressing progenitors, rarely produce RGCs (Hafler et al., 2012). Similarly, loss of Neurog1 increases early-born fates in the mouse cortex (Han et al., 2018), suggesting neurog1 crispants may similarly increase RGCs as they are one of the earliest born neurons in the retina. The effect of sox2 disruption may be dosage dependent, with low levels of expression promoting precocious RGC differentiation via upregulation of atoh7 (formerly ath5) and inhibition of Notch (Taranova et al., 2006). Constitutive expression of Ascl1 in mouse MG cells may actively repress RGC differentiation, thus Ascl1-independent means of dedifferentiating MG and/or methods for downregulating Ascl1 after MG dedifferentiation, may be required to promote RGC maturation. Regarding Ascl1-independent dedifferentiation, several other neurogenic factors, including sox2, sox10, neurod1, and neurog2, are able to reprogram astroglia cells into neurons (Talifu et al., 2022), as per overexpression of Ascl1 in mouse MG cells.

Minor:

1. There is limited reporting on the number of biological samples used in most of the statistical analysis used in this paper and the description of the statistical methodology is limited. For example, in figures 1 b, e, g, j, and l. Access to raw data is highly recommended. In supplemental fig S1b- it would be best to describe what the comparisons are since there are two bars with significance or ns. Directing the reader on what is being compared would be helpful. **** not defined in fig legend 1 and no description of the significance values on legend for Fig S1.

Response: 1) Sample sizes are now clarified in updated figure legends and/or supplemental Tables providing full statistical profiles for data points (sample size, effect size, p-value, and 95% confidence interval). 2) Raw data files are now provided with the resubmission materials. 3) The Fig. S1b graphic and legend was updated to show that all experimental conditions were compared to the two control conditions used to establish a 100% to 0% effect size range, non-ablated Cntrl and non-tg fish, respectively. 4) Definitions for asterisks are provided in an updated figure legend.

2. Figure 2C: Color coded lines for BC, Rods, HCs could be more obvious or more different than others to follow better

© 2024. Published by The Company of Biologists under the terms of the Creative Commons Attribution License (https://creativecommons.org/licenses/by/4.0/).

Response: Color codes for cell types were updated for clarity in the high-res resubmission version of Fig. 2c.

3. Some supplemental figures are not sharp enough to clearly read. Example, Fig S2- X and Y axis descriptions are blurry, especially the gene names.

Response: High-res versions of all figures are provided with the resubmission.

4. No description of how volcano plot was generated in Fig 2 and n values.

Response: A description of how volcano plots were generated is now included in the Materials and Methods. A new table was generated to provide p-values and fold-change info for all genes in the 12 hpa MG dataset.

5. Text within UMAPS like #s in subclustering – fig 2 or pseudotime UMAP text – fig 3a and fig 5e is hard to discern- need to be bolded or sharper.

Response: Have updated all figures to ensure legibility of labels.

6. Figure legend 3e should say MG>Progenitors> Rods not RGCs.

Response: Corrected.

7. Why are the plots of control RGC and Rod Controls not similar as these retinas were not ablated- Describe if there is a difference or why they are different.

Response: We assume this is in reference to Fig. 3h. The differences are due to one pseudotime trajectory being based on data for samples taken at 0, 12, 24, 48, and 72 hpa, versus 48 hpa alone. The figure has been updated to make this clearer.

8. Most figures do not describe the pvalues (*) in the legend, except Fig legend
1.

Response: We have added asterisk p-value definitions to all figure legends.

9. Label Fig S4 “d” with 5 dpf as “c” is labeled with 48 hpa for consistency

Response: We have updated x-axis conventions in order to maximize the size of data graphics and increase consistency of labeling.

10. There are few grammatical errors throughout. For example when referring to multiomic analysis- nuclei were isolated, not cells, therefore should be careful when referring to snRNA/ATACseq and not “sc”; ie page 7: profiling a total of 53,889 cells should be nuclei.

Response: Carefully checked and revised throughout to be consistent with assay type.

11. Fig 5b- Labels are too small to read, and also it would be nice to have the start site and gene direction to correlate it with the peaks reported (putative promoter/enhancer regions). There is no key color for the values on Fig 5 f and g.

Response: Updated Fig. 5b, increased font sizes and added genomic detail. Updated Fig. 5f with color code legend for gene expression changes.

12. Color code- red in Fig 5h is up-regulated but in 5i is down-regulated- it will be good to be consistent on color coding.

Response: Updated Fig. 5i to be consistent with color conventions, red = up-regulated, blue = down-regulated.

© 2024. Published by The Company of Biologists under the terms of the Creative Commons Attribution License (https://creativecommons.org/licenses/by/4.0/).

13. Ref figures on page 7 at the end of paragraph: “Neurogenic TFs upregulated in ascl1a KO progenitor cells included: crx, foxn4, neurod4, vsx1, otx2b, pou2f2a, and atoh7 – and all Sox genes evaluated, except sox2. Strong induction of sox11a/b and sox4a – both implicated in RGC development in mice(38) – suggests enhanced RGC fate bias in ascl1a KOs. Conversely, neurod1 and nrl showed reduced levels of expression in ascl1 KOs (Fig. 5g), suggesting reduced production of photoreceptors (Fig 5f and g).

Response: Revised, “Neurogenic TFs upregulated in ascl1a KO progenitor cells included: crx, foxn4, neurod4, vsx1, otx2b, pou2f2a, and atoh7 (Fig. 5f) and all Sox gene family members evaluated, except sox2 (Fig 5g). Strong induction of sox11a/b and sox4a (Fig. 5g) – both of which are implicated in RGC development in mice(38) – further supports enhanced RGC fate bias in ascl1a KOs. Conversely, neurod1 and nrl showed reduced levels of expression in ascl1 KOs (Fig. 5f), suggesting reduced production of photoreceptors.

14. In the first paragraph of page 9, “this could also account for the pro-RGC regenerative effects of knocking out olig2(49) and possibly neurog”. A reference for neurog is missing, and # 49 reference describe the cellular fate of embryonic Olig2+ retinal progenitor cells, and no knockout of olig2 was used. Other missing references are noted in the manuscript; therefore, it is advisable for the authors to carefully review all references for proper citation and inclusion.

Response: The referenced studies show that ascl1-expressing (Brzezinski et al., 2011) and olig2-expressing (Hafler et al., 2012) mouse retinal progenitor cells (RPCs) give rise to retinal cell types other than RGCs during development: all retinal cell types except RGCs for ascl1+ RPCS; rod, bipolar, amacrine, and MG cells for olig2+ RPCs. We therefore interpret the accelerated production of RGCs by regenerative RPCs in the absence ascl1a and olig2 to result from decreased probabilities of producing retinal cell types other than RGCs; i.e., removing pathways that give rise to non-RGCs. We elaborate further on this in the revised manuscript to make this point clearer:
“…Increased fate bias could also account for the pro-RGC regenerative effects of knocking out two other bHLH transcription factors, olig2 and neurog1. Olig2-expressing mouse retinal progenitors, like Ascl1-expressing progenitors, rarely produce RGCs (Hafler et al., 2012). Similarly, loss of Neurog1 increases early-born fates in the mouse cortex (Han et al., 2018), suggesting neurog1 crispants may similarly increase RGCs as they are one of the earliest born neurons in the retina. The effect of sox2 disruption may be dosage dependent, with low levels of expression promoting precocious RGC differentiation via upregulation of atoh7 (formerly ath5) and inhibition of Notch (Taranova et al., 2006).

15. define dpa

Response: Days post-ablation (dpa) was erroneously defined at the second instance in the initial submission, it is now defined at the first instance, “Co-immunostaining assays performed at 1 day post-fertilization (dpa) demonstrated colocalization of PCNA and glutamine synthetase (GS), a marker of MG cells (fig. S1e).”

<u>Second decision letter</u>

MS ID#: DEVELOP/2024/202754

MS TITLE: Large-scale CRISPR screen reveals context-specific genetic regulation of retinal ganglion cell regeneration

AUTHORS: Kevin Emmerich, John Hageter, Thanh Hoang, Pin Lyu, Abigail V Sharrock, Anneliese Ceisel, James Thierer, Zeeshaan Chunawala, Saumya Nimmagadda, Isabella Palazzo, Frazer Matthews, Liyun Zhang, David T White, Catalina Rodriguez, Gianna Graziano, Patrick Marcos, Adam May, Tim Mulligan, Barak Reibman, Meera T Saxena, David F Ackerley, Jiang Qian, Seth Blackshaw, Eric Horstick, and Jeff S Mumm

© 2024. Published by The Company of Biologists under the terms of the Creative Commons Attribution License (https://creativecommons.org/licenses/by/4.0/).

I am happy to tell you that your manuscript has been accepted for publication in Development, pending our standard publication integrity checks.

© 2024. Published by The Company of Biologists under the terms of the Creative Commons Attribution License (https://creativecommons.org/licenses/by/4.0/).
